# 1399 H&E-stained sentinel lymph node sections of breast cancer patients: the CAMELYON dataset

**DOI:** 10.1093/gigascience/giy065

**Published:** 2018-05-31

**Authors:** Geert Litjens, Peter Bandi, Babak Ehteshami Bejnordi, Oscar Geessink, Maschenka Balkenhol, Peter Bult, Altuna Halilovic, Meyke Hermsen, Rob van de Loo, Rob Vogels, Quirine F Manson, Nikolas Stathonikos, Alexi Baidoshvili, Paul van Diest, Carla Wauters, Marcory van Dijk, Jeroen van der Laak

**Affiliations:** 1 Diagnostic Image Analysis Group, Department of Pathology, Radboud University Medical Center, Huispost 824, Geert Grootteplein-Zuid 10, 6525GA Nijmegen, The Netherlands; 2 Department of Pathology, University Medical Center Huispost H04.312, Heidelberglaan 100, 3584CX, Utrecht, The Netherlands; 3 Laboratory for Pathology East Netherlands (LabPON), Postbus 516, 7550AM Hengelo, The Netherlands; 4 Department of Pathology, Canisius-Wilhelmina Hospital, Postbus 9015, 6500GS Nijmegen, The Netherlands; 5 Department of Pathology, Rijnstate Hospital, Pathology-DNA, Postbus 9555, 6800TA Arnhem, The Netherlands

**Keywords:** breast cancer, lymph node metastases, whole-slide images, grand challenge, sentinel node

## Abstract

**Background:**

The presence of lymph node metastases is one of the most important factors in breast cancer prognosis. The most common way to assess regional lymph node status is the sentinel lymph node procedure. The sentinel lymph node is the most likely lymph node to contain metastasized cancer cells and is excised, histopathologically processed, and examined by a pathologist. This tedious examination process is time-consuming and can lead to small metastases being missed. However, recent advances in whole-slide imaging and machine learning have opened an avenue for analysis of digitized lymph node sections with computer algorithms. For example, convolutional neural networks, a type of machine-learning algorithm, can be used to automatically detect cancer metastases in lymph nodes with high accuracy. To train machine-learning models, large, well-curated datasets are needed.

**Results:**

We released a dataset of 1,399 annotated whole-slide images (WSIs) of lymph nodes, both with and without metastases, in 3 terabytes of data in the context of the CAMELYON16 and CAMELYON17 Grand Challenges. Slides were collected from five medical centers to cover a broad range of image appearance and staining variations. Each WSI has a slide-level label indicating whether it contains no metastases, macro-metastases, micro-metastases, or isolated tumor cells. Furthermore, for 209 WSIs, detailed hand-drawn contours for all metastases are provided. Last, open-source software tools to visualize and interact with the data have been made available.

**Conclusions:**

A unique dataset of annotated, whole-slide digital histopathology images has been provided with high potential for re-use.

## Background

Breast cancer is one of the most common and deadly cancers in women worldwide [1]. Although prognosis for breast cancer patients is generally good, with an average5-year overall survival rate of 90% and 10-year survival rate of 83%, it significantly deteriorates when breast cancer metastasizes [2]. While localized breast cancer has a five-year survival rate of 99%, this drops to 85% in the case of regional (lymph node) metastases and only 26% in case of distant metastases. As such, it is of the utmost importance to establish whether metastases are present to allow adequate treatment and the best chance of survival. This is formally captured in the tumor, node, metastasis (TNM) staging criteria [3].

The first step in determining the presence of metastases is to examine the regional lymph nodes. Not only is the presence of metastases in these lymph nodes a poor prognostic factor by itself, it is also an important predictive factor for the presence of distant metastases [4]. In breast cancer, the most common way to assess the regional lymph node status is the sentinel lymph node procedure [5, 6]. With this procedure, a blue dye and/or radioactive tracer is injected near the tumor. The first lymph node reached by the injected substance, the sentinel node, is most likely to contain the metastasized cancer cells and is excised. Subsequently, it is submitted for histopathological processing and examination by a pathologist.

**Figure 1:**
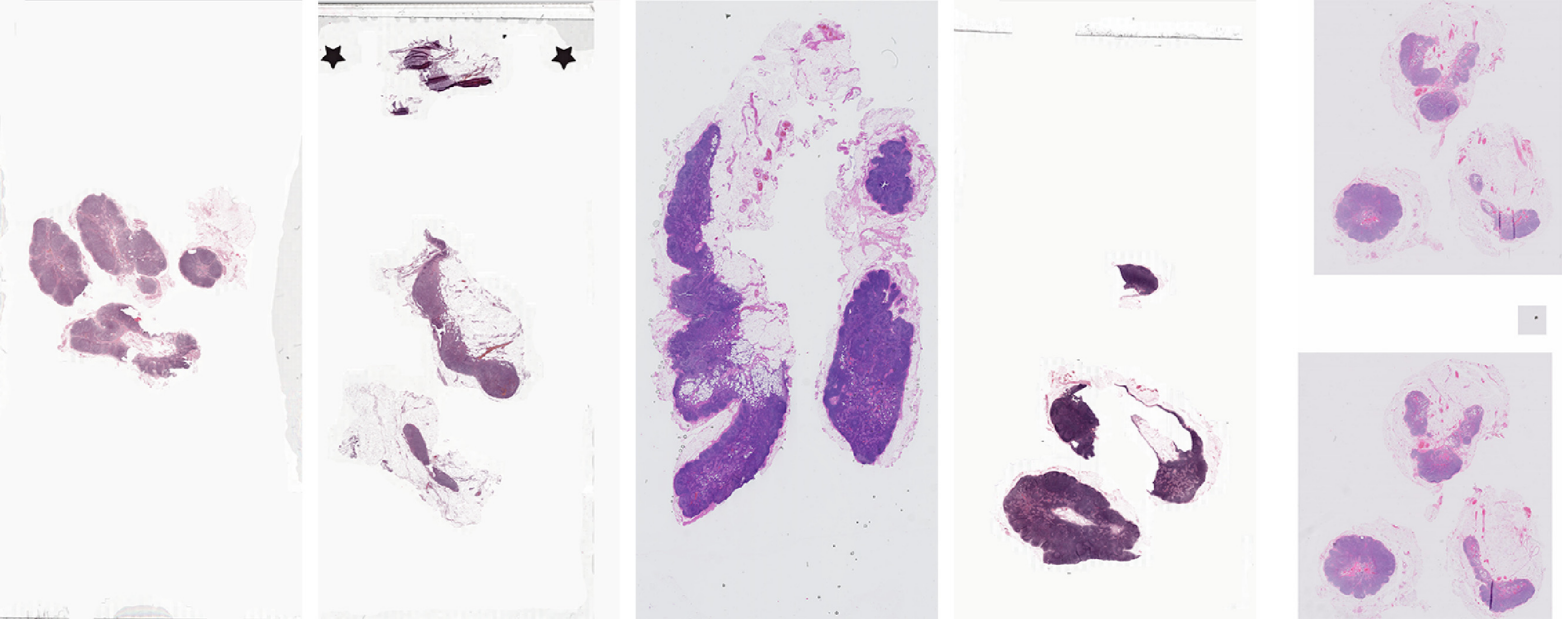
Low-resolution example of a WSI from each of the five centers contributing data.

The pathologist examines a glass slide containing a tissue section of the lymph node stained with hematoxylin and eosin (H&E). Examples are shown in Fig 1.Based on solitary tumor cells or the diameter of clusters of tumor cells, metastases can be divided into one of three categories: macro-metastases, micro-metastases, or isolated tumor cells (ITC). The size criteria for each of these categories is shown in Table1. Based on the presence or absence of one or more of these metastasis, an initial pathological N-stage (pN-stage) is assigned to a patient. Based on this initial stage, in combination with characteristics of the main tumor, further lymph node dissection or axillary radiotherapy may be performed. These axillary lymph nodes are then also pathologically assessed to come to a final pN-stage. pN categorization is mostly based on metastasis size and the number of lymph nodes involved but also on the anatomical location of the lymph nodes. A small excerpt of the pN stage is shown in Table 2; for a full listing, refer to the 7th edition of the TNM staging criteria for breast cancer [7].

**Table 1:** Rules for assigning clusters of metastasized tumor cells to a metastasis category

Category	Size
Macro-metastasis	Larger than 2 mm
Micro-metastasis	Larger than 0.2 mm and/or containing more than 200 cells, but not larger than 2 mm
Isolated tumor cells	Single tumor cells or a cluster of tumor cells not larger than 0.2 mm or less than 200 cells

**Table 2:** Selection of N-stages for staging of breast cancer based on the 7th edition of the TNM staging criteria

Stage	Description
N0	Cancer has not spread to nearby lymph nodes
N0(i+)	Lymph nodes only contain ITCs
N1mi	Micro-metastases in 1 to 3 lymph nodes axillary
N1a	Cancer has spread to 1 to 3 lymph nodes axillary, with at least 1 macro-metastasis
N1b	Cancer has spread to internal mammary lymph nodes, but this spread could only be found on sentinel lymph node biopsy
N1c	Both N1a and N1b apply
N2a	Cancer has spread to 4 to 9 lymph nodes under the arm, with at least 1 macro-metastasis
N2b	Metastases in clinically detected internal mammary lymph nodes in the absence of axillary lymph node metastases

**Figure 2:**
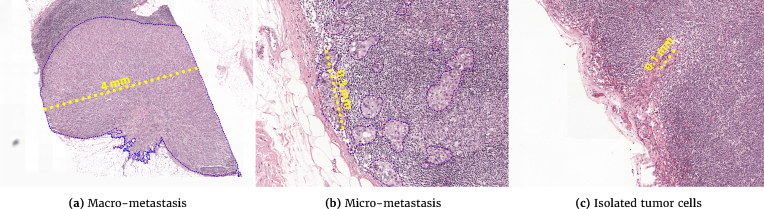
Representative samples of the different sizes of breast cancer metastases in sentinel lymph nodes.

A key challenge for pathologists in assessing lymph node status is the large area of tissue that has to be examined to identify metastases that can be as small as single cells. Examples of a macro-metastasis, micro-metastasis, and ITC are shown in Fig 1 and Fig. 2. For sentinel lymph nodes, at least three sections at different levels through the lymph node have to be examined; for non-sentinel lymph nodes, one section of at least 10 lymph nodes has to be examined [8, 9]. This tedious examination process is time-consuming, and pathologists may miss small metastases [10]. In the Netherlands, a secondary examination using an immunohistochemical staining for cytokeratin has to be performed if inspection of the H&E slide identifies no metastases. However, even in this secondary examination, metastases can still be missed [11].

Today, advances in whole-slide imaging and machine learning have opened an avenue for analysis of digitized lymph node sections with computer algorithms. Whole-slide imaging is a technique where high-speed slide scanners digitize glass slides at very high resolution (e.g., 240 nm per pixel). This results in images with a size on the order of 10 gigapixels, typically called whole-slide images (WSIs). This large amount of data makes WSIs ideally suited for analysis with machine-learning algorithms. Although machine -earning algorithms have been applied to digitized pathology data as early as 1994 [12], WSIs have only appeared since early 2000. Since then, many researchers have described the use of machine-learning algorithms in WSIs, e.g., for breast or prostate cancer classification [13, 14]. Over the past five years, so-called deep learning algorithms, such as convolutional neural networks (CNNs), have become incredibly popular. For example, we were the first to show that training CNNs to detect cancer metastases in lymph nodes was possible and potentially could result in improved efficiency and accuracy of histopathologic diagnostics [15].

To train machine-learning models, large, well-curated datasets are needed to both train these models and accurately evaluate their performance. To allow the broader computer vision community to replicate and build on our results, we publicly released a large dataset of annotated WSIs of lymph nodes, both with and without metastases in the context of the CAMELYON16 and CAMELYON17 challenges (CAncer MEtastases in LYmph nOdes challeNge) [16, 17].

The concept of challenges in medical imaging and computer vision has been around for nearly a decade. In medical imaging it primarily started with the liver segmentation challenge at the annual MICCAI conference in 2007 [18], and in computer vision, the ImageNet Challenge is most widely known [19]. The main goal of challenges, both in medical imaging and in computer vision, is to allow a meaningful comparison of algorithms. In scientific literature, this was often not the case as authors present results on their own, often proprietary, datasets with their own choice of evaluation metrics. In medical imaging, this was specifically a problem as sharing medical data is often difficult. Challenges change this by making available datasets and enforcing standardized evaluation. Furthermore, challenges have the added benefit of opening up meaningful research questions to a large community who normally might not have access to the necessary datasets.

The CAMELYON dataset was collected at different Dutch medical centers to cover the heterogeneity encountered in clinical practice. It contains 1,399 WSIs, resulting in approximately 3 terabytes of image data. We released a part of the dataset with the reference standard (i.e., the training set) to allow other groups to build algorithms to detect metastases. Subsequently, the rest of the dataset was released without a reference standard (i.e., the test set). Participating teams could submit their algorithm output on the test set to us, after which we evaluated their performance on a predefined set of metrics to allow fair and standardized comparison to other teams. To enable participation of teams that are not familiar with WSIs, we released a publicly available software package for viewing WSIs, annotations, and algorithmic results, dubbed the automated slide analysis platform (ASAP) [20].

Here, we describe the CAMELYON dataset in detail and cover the following topics:

- Sample collection
- Slide digitization and conversion
- Challenge dataset construction and statistics
- Instructions on the use of ASAP to view and analyze slides
- Suggestions for data re-use

## Data description

The CAMELYON dataset is a combination of the WSIs of sentinel lymph node tissue sections collected for the CAMELYON16 and CAMELYON17 challenges, which contained 399 WSIs and 1,000 WSIs, respectively. This resulted in 1399 unique WSIs and a total data size of 2.95 terabytes. The dataset is currently publicly available after registration via the CAMELYON17 website [17]. At the time of writing, it had been accessed by more than 1,000 registered users worldwide. It has been licensed under the Creative Commons CC0 license.

### Data collection

Collection of the data was approved by the local ethics committee of the Radboud University Medical Center (RUMC) under 2016-2761, and the need for informed consent was waived. Data were collected at five medical centers in the Netherlands: the RUMC, the Utrecht University Medical Center (UMCU), the Rijnstate Hospital (RST), the Canisius-Wilhelmina Hospital (CWZ), and LabPON (LPON). An example of digitized slides from these centers can be seen in Fig.1.

Initial identification of cases eligible for inclusion was based on local pathology reports of sentinel lymph node procedures between 2006 and 2016. The exact years varied from center to center but did not affect data distribution or quality. After the lists of sentinel node procedures and the corresponding glass slides containing H&E-stained tissue sections were obtained, slides were randomly selected for inclusion. As the vast majority of sentinel lymph nodes are negative for metastases, selection was stratified for the presence of macro-metastases, micro-metastases, and ITCs based on the original pathology reports. This was done to obtain a good representation of differing metastasis appearance without the need for an excessively large dataset.

Data were acquired in two stages, corresponding to the time periods for organization of the CAMELYON16 and CAMELYON17 challenges. Within the CAMELYON16 challenge, only data from the RUMC and UMCU were acquired, and no slides containing only ITCs were included. For CAMELYON17, data were included from all five centers, and glass slides containing only ITCs were obtained as well. A categorization of the slides can be found in Tables 3 and 4.

**Table 3:** WSI-level characteristics for the CAMELYON16 part of the dataset

		Metastases		
Center	Total WSIs	None	Macro	Micro
RUMC	249	150	48	51
UMCU	150	90	34	26

**Table 4:** WSI-level characteristics for the CAMELYON17 part of the dataset

Center	Total WSIs		Metastases (Train)			
	Train	Test	None	Macro	Micro	ITC
CWZ	100	100	64	15	10	11
LPON	100	100	64	25	4	7
RST	100	100	60	11	22	7
RUMC	100	100	60	19	13	8
UMCU	100	100	75	15	8	2
Total	500	500	323	85	57	35

**Table 5:** Patient-level characteristics for the CAMELYON17 part of the dataset

Center	Total patients		Stages (Train)				
	Train	Test	pN0	pN0_i +_	pN1_mi_	pN1	pN2
CWZ	20	20	4	3	5	7	1
LPON	20	20	6	2	2	7	3
RST	20	20	4	2	6	5	3
RUMC	20	20	3	2	4	8	3
UMCU	20	20	8	2	4	3	3
Total	100	100	25	11	21	30	13

After glass slides were selected, they were digitized with different slide scanners such that scan variability across centers was captured in addition to H&E staining procedure variability. The slides each from RUMC, CWZ, and RST were scanned with the 3DHistech Pannoramic Flash II 250 scanner at the RUMC. At the UMCU, slides were scanned with a Hamamatsu NanoZoomer-XR C12000-01 scanner, and at LPON with a Philips Ultrafast Scanner.

As all slides are initially stored in an original vendor format that makes re-use challenging, slides were converted to a common, generic TIFF (tagged image file format) using an open-source file converter, part of the ASAP package [20]. As there are no open-source tools to convert the iSyntax format produced by the Philips Ultrafast Scanner, a proprietary converter was used to convert files to a special TIFF format [21] that can be read by the open-source package OpenSlide [22] and the ASAP package [20]. Some basic descriptors are shown in Table 6.

**Table 6:** Basic descriptors for the TIFF used in the CAMELYON dataset

Format	Tiled TIFF (bigTIFF)
Tile size	512 pixels
Pixel resolution	0.23 μm to 0.25 μm
Channels per pixel	3 (red, green, blue)
Bits per channel	8
Data type	Unsigned char
Compression	JPEG

After digitization, the reference standard for each slide needed to be established. The reference standard for each WSI consisted of a slide-level label indicating the largest metastasis within a slide (i.e., no metastasis, macro-metastasis, micro-metastasis, or ITC). In addition, for all 399 WSIs that were part of the CAMELYON16 challenge and an additional 50 WSIs from the CAMELYON17 challenge, detailed contours were drawn along the boundaries of metastases within the WSI. For the 50 slides of the CAMELYON17 challenge, 10 slides from each center were used to allow users of the dataset to analyze metastasis appearance differences across different centers.

Initial slide-level labels were assigned based on the pathology reports obtained from clinical routine. For the CAMELYON16 part of the dataset, all slides were subsequently examined and metastases outlined by an experienced lab technician (M.H.) and a clinical PhD student (Q.M.). Afterward, all annotations were inspected by one of two expert breast pathologists (P.B. or P.v.D.). Some slides contained two consecutive tissue sections of the same lymph node, in which case only one of the two sections was annotated as this did not affect the slide-level label. In total, 15 slides may contain unlabeled metastatic areas and are indicated via a descriptive text file that is part of the dataset.

For the CAMELYON17 part of the dataset, an experienced general pathologist (M.v.D.) inspected all the slides to assess the slide-level labels. For the 50 slides with detailed annotations, experienced observers (M.v.D., M.H., Q.M., O.G., and R.vd.L.) annotated all metastases. Subsequently, these annotations were double-checked by one of the other observers or one of two pathology residents (A.H. and R.V.).

**Figure 3:**
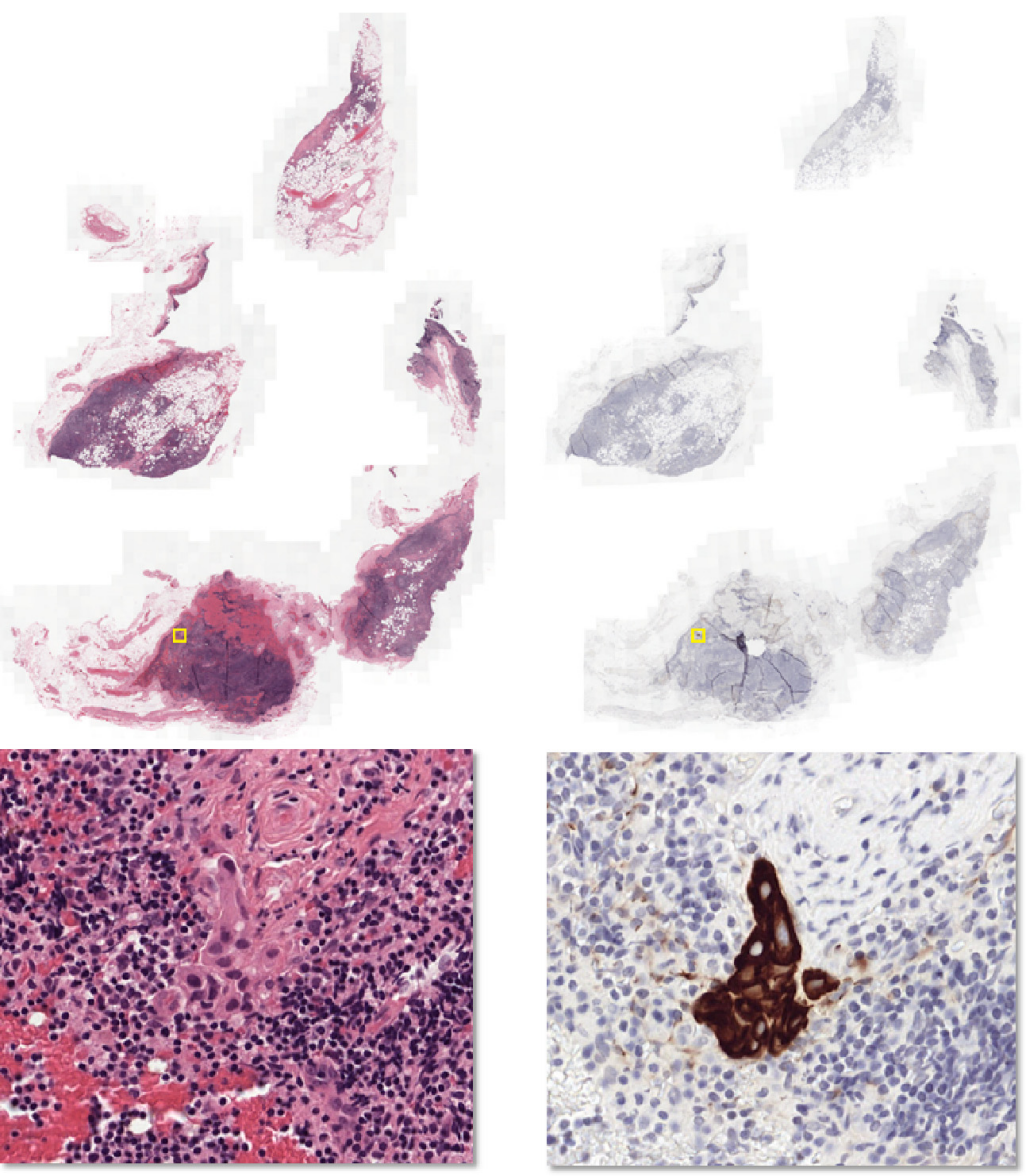
H&E-stained tissue section and a consecutive section immunohistochemically stained for cytokeratin. The top row shows the low-resolution images and the bottom row a high-resolution image, centered at a metastasis. The metastasis is difficult to see in H&E but easy to identify in the immunohistochemically stained slide. A yellow bounding box indicates the metastasis location in the images in the top row.

For the entire dataset, when the slide-level label was unclear during the inspection of the H&E-stained slide, an additional WSI with a consecutive tissue section, immunohistochemically stained for cytokeratin, was used to confirm the classification. Furthermore, this stain was also used to aid in drawing the outlines in both CAMELYON16 and CAMELYON17, which helps limit observer variability. As both the H&E and IHC slides are digital, they can be viewed simultaneously, allowing observers to easily identify the same areas in both slides. This stain is also used in daily clinical pathology practice to resolve diagnosis in the case of metastasis-negative H&E [23, 24]. An example of an H&E WSI and the corresponding consecutive cytokeratin immunohistochemical section are shown in Fig.3.

In the CAMELYON17 dataset, after establishing the reference standard, slides were divided into artificial patients, covering the different pN-stages (see Table 2). Each artificial patient only had WSIs from one center. For each artificial patient in the training part of the dataset, the pN-stage and the slide-level labels were provided. This was done to assess the potential of participating algorithms within the challenge to perform automated pN-staging. However, all WSIs can be used independently of their patient-level labels. A complete overview of the patient-level characteristics is shown in Table 5.

After the dataset and reference standard were established, we uploaded the entire dataset to Google Drive and to BaiduPan. These two options were chosen to reach as wide an audience as possible, given that Google Drive is not accessible everywhere (e.g., People’s Republic of China). A link to the data was shared with participants after registration at the CAMELYON websites [16, 17].

### Data validation and quality control

All glass slides included in the CAMELYON dataset were part of routine clinical care and are thus of diagnostic quality. However, during the acquisition process, scanning can fail or result in out-of-focus images. As a quality-control measure, all slides were inspected manually after scanning. The inspection was performed by an experienced technician (Q.M. and N.S. for UMCU, M.H. or R.vd.L. for the other centers) to assess the quality of the scan; when in doubt, a pathologist was consulted on whether scanning issues might affect diagnosis.

Due to the inclusion of IHC for establishing the reference standard, the chance of errors being made can be considered limited, as pathologists make few mistakes in identifying metastases with IHC [25]. Furthermore, all slides were checked twice. However, to further ensure the quality of the reference standard, we looked at algorithmic results submitted to the challenge to identify slides where the best performing algorithms disagreed with the reference standard. This led to a correction of the reference standard in 3 of the 1,399 slides.

### Tools for data use

Several tools are available to visualize and interact with the CAMELYON dataset. Here, we present examples of how to use the data with an open-source package developed by us, ASAP [20]. Other open-source packages are also available, such as OpenSlide [26], but those do not contain functionality for reading annotations or storing image analysis results.

- Project name: Automated Slide Analysis Platform (ASAP)
- Project home page: https://github.com/GeertLitjens/ASAP
- Operating system(s): Linux, Windows
- Programming language: C++, Python
- Other requirements: CMake (www.cmake.org)
- License: GNU GPL v2.0

**Figure 4:**
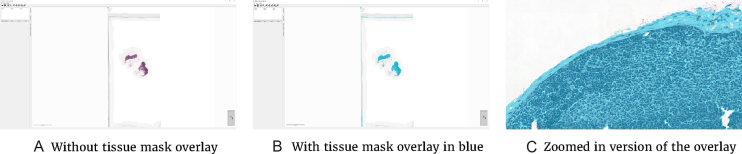
Interface of the ASAP viewer interface. Visible items are the annotations tools in toolbar, the viewport showing the WSI, and the plugin panel on the left.

ASAP contains several components, of which one is a viewer/annotation application (Fig. 4). This can be started via the ASAP executable within the installation folder of the package. After opening an image file from the CAMELYON dataset, one can explore the data via a Google Maps-like interface. The provided reference standard can be loaded via the annotation plugin. In addition, new annotations can be made with the annotation tools provided. Last, the viewer is not limited to files from the CAMELYON dataset but can visualize most WSI formats.

In addition to the viewer application and C++ library for reading and writing WSI images, we also provide Python-wrapped modules. To access the data via Python, the following code snippet can be used.

**Figure:**
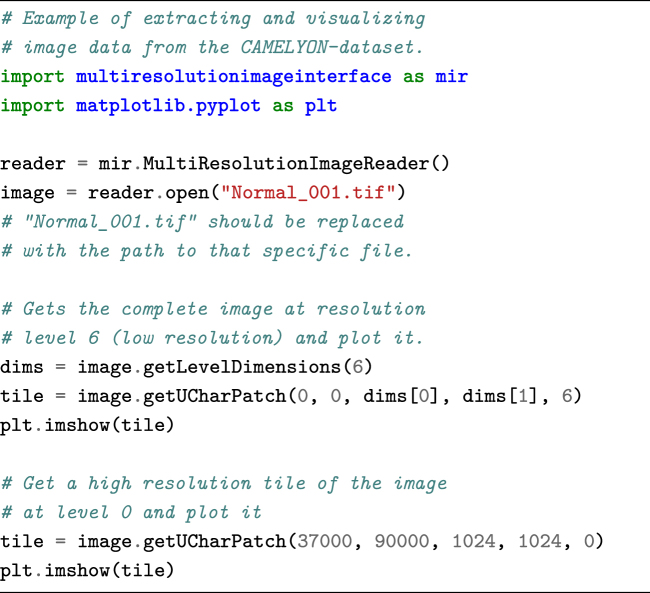


The annotations are provided in human-readable XML format and can be parsed using the ASAP package. However, other XML reading libraries can also be used. Annotations are stored as polygons. Each polygon consists of a list of (x, y) coordinates at the highest resolution level of the image. Annotations can be converted to binary images via the following code snippet.

**Figure:**
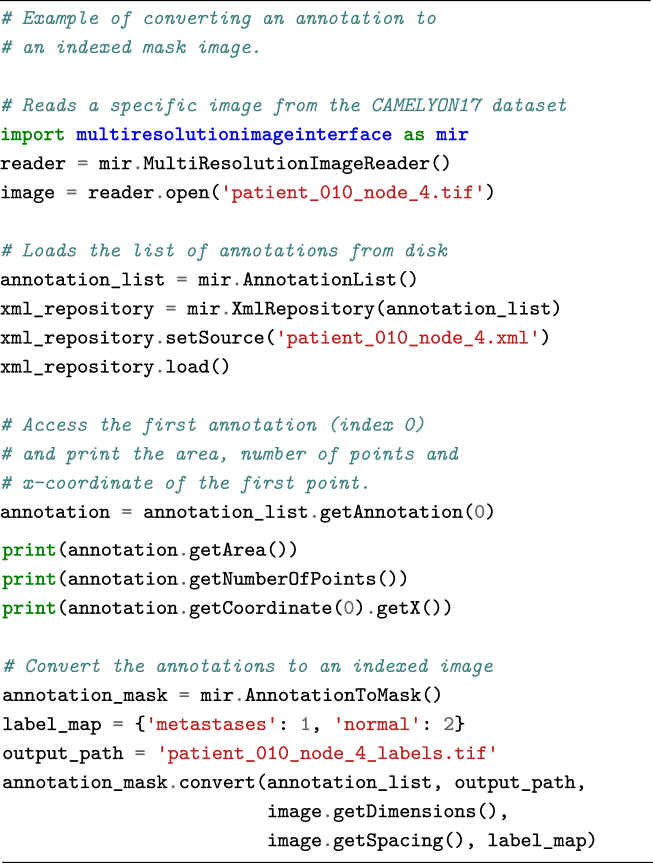


The Python package can also be used to perform image processing or machine-learning tasks on the data and to write out an image result. The code snippet below performs some basic thresholding to generate a background mask. These results can then subsequently be visualized using the viewer component of ASAP, which also supports floating point images. An example of the code snippet result can be seen in Fig.4B.

**Figure:**
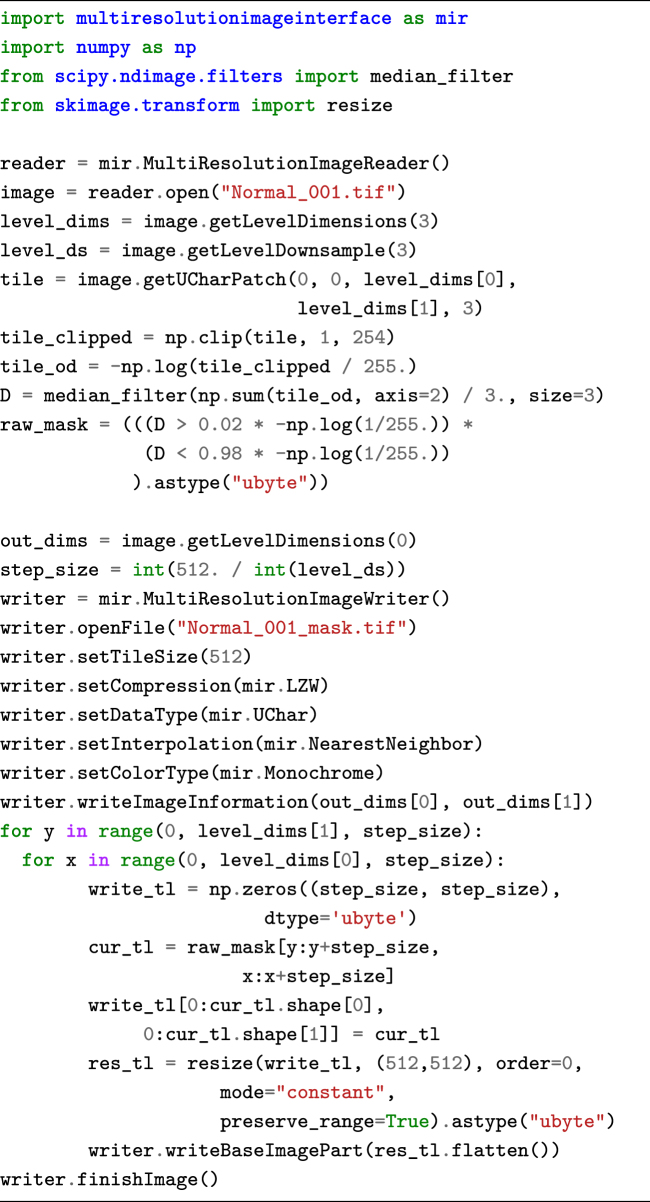


The ASAP package also supports writing your own image processing routines and integrating them as plugins into the viewer component. Some existing examples such as color deconvolution and nuclei detection are provided.

### Re-use potential

The CAMELYON dataset is currently being used within the CAMELYON17 challenge, which is open for new participants and submissions. In this context, the dataset enables testing of new machine-learning and image analysis strategies against the current state-of-the-art. Within CAMELYON, we evaluate the algorithms based on a weighted Cohen’s kappa at the pN-stage level [27]. This statistic measures the categorical agreement between the algorithm and the reference standard where a value of 0 indicates agreement at the level of chance and 1 is perfect agreement. The quadratic weighting penalizes deviations of more than one category more severely. Conclusions arising from such experiments may have significance for the broader field of computational pathology, rather than being restricted to this particular application. For example, experiments with weakly supervised machine learning in histopathology may benefit from the CAMELYON dataset, with an established baseline based on fully supervised machine learning.

The dataset has also been used by companies experienced in machine-learning applications to be a first foray into digital pathology, e.g., Google [28]. Because of its extent, observer experiments with pathologists may be performed to assess the value of algorithms within a diagnostic setting. For example, a comparison of algorithms competing in the CAMELYON16 challenge to pathologists in clinical practice was recently published [29]. Experiments with the dataset may serve to identify relevant issues with implementation, validation, and regulatory affairs with respect to computational pathology.

A key example of implementation issues with respect to machine-learning algorithms in medical imaging is generalization to different centers. In pathology, centers can differ in tissue preparation, staining protocol, and scanning equipment. This can have a profound impact on image appearance. In the CAMELYON dataset, we included data from five centers and three scanners. We are confident that algorithms trained with this data will generalize well. Users of the dataset can even explicitly evaluate this as we have indicated for each image the center from which it was obtained. By leaving out one center and evaluating performance on that center specifically, the participants can assess the robustness of their algorithms.

We believe the usefulness of the dataset also extends beyond its initial use within the CAMELYON challenge. For example, it can be used for evaluation of color normalization algorithms and for cell detection/segmentation algorithms.

## Availability of supporting data

CAMELYON16 and CAMELYON17 datasets are open access and shared publicly via the CAMELYON17 [17] website. Snapshots of this data and the code of ASAP [20] are also hosted in the *GigaScience* GigaDB database [30].

## Abbreviations

ASAP: automated slide analysis platform; CNN: convolutional neural network; CWZ: anisius-Wilhelmina Hospital; H&E: hematoxylin and eosin; IHC: immunohistochemistry; ITC: isolated tumor cell; LPON: LabPON; pN-stage: pathological N-stage; RST: Rijnstate Hospital; RUMC: Radboud University Medical Center; TIFF: tagged image file format; TNM: tumor, node, metastasis; UMCU: Utrecht University Medical Center; WSI: whole-slide image

## Ethical approval

The collection of the data was approved by the local ethics committee (Commissie Mensgebonden Onderzoek regio Arnhem - Nijmegen) under 2016-2761, and the need for informed consent was waived.

## Competing interests

JvdL, PvD, and AB are members of the scientific advisory board of Philips Digital Pathology (Best, The Netherlands). JvdL is also part of the scientific advisory board of ContextVision (Stockholm, Sweden), and PvD is part of the scientific advisory board of Sectra (Linköping, Sweden).

## Funding

Data collection and annotation where funded by Stichting IT Projecten and by the Fonds Economische Structuurversterking (tEPIS/TRAIT project; LSH-FES Program 2009; DFES1029161 and FES1103JJTBU). This work was also supported by grant 601040 from the FP7-funded VPH-PRISM project of the European Union.

## Author Contributions

GL and JvdL designed the study and supervised the collection of the dataset. GL wrote the initial draft and final version of the manuscript. PBu, OG, BEB, MB, MH, QM, AB, NS, PvD, MvD, and CW were involved in sample collection. GL, PBa, and NS were involved in data anonymization and conversion. PBu, OG, MH, MB, MvD, QM, AH, RV, and PvD were involved in establishing the reference standard. All authors were involved in reviewing and finalizing the paper.

## Supplementary Material

GIGA-D-17-00346_Original_Submission.pdf

GIGA-D-17-00346_Revision_1.pdf

GIGA-D-17-00346_Revision_2.pdf

Response_to_Reviewer_Comments_Original_Submission.pdf

Response_to_Reviewer_Comments_Revision1.pdf

Reviewer_1_Report_(Original_Submission) -- Lior Shamir 1/3/2018 Reviewed

Reviewer_1_Report_(Revision_1) -- Lior Shamir 3/30/2018 Reviewed

Reviewer_2_Report_(Original_Submission) -- Chris Armit 1/4/2018 Reviewed

Reviewer_2_Report_(Revision_1) -- Chris Armit 02-04-2018 Reviewed

Supplement Files
