# Supplementary material for: 1399 H&E-stained sentinel lymph node sections of breast cancer patients: the CAMELYON dataset
Source: Gigascience. 2018 May 31;7(6):giy065. doi: 10.1093/gigascience/giy065 (PMC6007545; doi:10.1093/gigascience/giy065)
Supplement: GIGA-D-17-00346_Original_Submission.pdf [file giy065_giga-d-17-00346_original_submission.pdf]

GigaScience

1399 H&E-stained sentinel lymph node sections of breast cancer patients: the CAMELYON dataset

--Manuscript Draft--

Manuscript Number:	GIGA-D-17-00346
Full Title:	1399 H&E-stained sentinel lymph node sections of breast cancer patients: the CAMELYON dataset
Article Type:	Data Note
Funding Information:	Fonds Economische Structuurversterking (DFES1029161) — Not applicable<br>Stichting IT Projecten — Dr Jeroen van der Laak<br>FP7 Ideas: European Research Council () (601040) — Not applicable

Abstract:

Background

The presence of lymph node metastases is one of the most important factors in breast cancer prognosis. The most common strategy to assess the regional lymph node status is the sentinel lymph node procedure. The sentinel lymph node is the most likely lymph node to contain metastasized cancer cells and is excised, histopathologically processed and examined by the pathologist. This tedious examination process is time-consuming and can lead to small metastases being missed. However, recent advances in whole-slide imaging and deep learning have opened an avenue for analysis of digitized lymph node sections with computer algorithms. Convolutional neural networks, a type of deep learning algorithm, are able to automatically detect cancer metastases in lymph nodes with high accuracy. To train deep learning models, large, well-curated datasets are needed.

Results

We released a dataset of 1399 annotated whole-slide images of lymph nodes, both with and without metastases, in total three terabytes of data. Slides were collected from five different medical centers to cover a broad range of image appearance and staining variations. Each whole-slide image has a slide-level label indicating whether it contains no metastases, macro-metastases, micro-metastases or isolated tumor cells. Furthermore, for 209 whole-slide images, detailed hand-drawn contours for all metastases are provided. Last, open-source software tools to visualize and interact with the data have been made available.

Conclusions

A unique dataset of annotated, whole-slide digital histopathology images has been provided with high potential for re-use.

Corresponding Author:	Geert Litjens<br><br>NETHERLANDS
Corresponding Author Secondary Information:	
Corresponding Author's Institution:	
Corresponding Author's Secondary Institution:	
First Author:	Geert Litjens
First Author Secondary Information:	
Order of Authors:	Geert Litjens
	Peter Bandi

	Babak Ehteshami Bejnordi
	Oscar Geessink
	Maschenka Balkenhol
	Peter Bult
	Altuna Halilovic
	Meyke Hermsen
	Rob van de Loo
	Rob Vogels
	Quirine Manson
	Nikolas Stathonikos
	Alexi Baidoshvili
	Paul van Diest
	Carla Wauters
	Marcory van Dijk
	Jeroen van der Laak
**Order of Authors Secondary Information:**	
**Opposed Reviewers:**	
**Additional Information:**	
**Question**	**Response**
Are you submitting this manuscript to a special series or article collection?	No
**Experimental design and statistics**<br><br>Full details of the experimental design and statistical methods used should be given in the Methods section, as detailed in our Minimum Standards Reporting Checklist. Information essential to interpreting the data presented should be made available in the figure legends.<br><br>Have you included all the information requested in your manuscript?	Yes
**Resources**<br><br>A description of all resources used, including antibodies, cell lines, animals and software tools, with enough information to allow them to be uniquely identified, should be included in the Methods section. Authors are strongly encouraged to cite Research Resource Identifiers (RRIDs) for antibodies, model organisms and tools, where possible.	Yes

Powered by Editorial Manager® and ProduXion Manager® from Aries Systems Corporation

Have you included the information requested as detailed in our Minimum Standards Reporting Checklist?	
**Availability of data and materials**<br><br>All datasets and code on which the conclusions of the paper rely must be either included in your submission or deposited in publicly available repositories (where available and ethically appropriate), referencing such data using a unique identifier in the references and in the “Availability of Data and Materials” section of your manuscript.<br><br>Have you have met the above requirement as detailed in our Minimum Standards Reporting Checklist?	Yes

Powered by Editorial Manager® and ProduXion Manager® from Aries Systems Corporation

Manuscript

Click here to download Manuscript CAMELYON_GigaScience.pdf

OXFORD (GIGA)^n SCIENCE

GigaScience, 2017, 1–7

doi: xx.xxxx/xxxx
Manuscript in Preparation
Data Note

DATA NOTE

1399 H&E-stained sentinel lymph node sections of breast cancer patients: the CAMELYON dataset

Geert Litjens[1,*], Peter Bandi[1,†], Babak Ehteshami Bejnordi[1,†], Oscar Geessink[1†], Maschenka Balkenhol[1], Peter Bult[1], Altuna Halilovic[1], Meyke Hermsen[1], Rob van de Loo[1], Rob Vogels[1], Quirine F. Manson[2], Nikolas Stathonikos[2], Alexi Baidoshvili[3], Paul van Diest[2], Carla Wauters[4], Marcory van Dijk[5] and Jeroen van der Laak[1]

[1]Diagnostic Image Analysis Group, Department of Pathology, Radboud University Medical Center, Nijmegen, The Netherlands and [2]Department of Pathology, University Medical Center Utrecht, Utrecht, The Netherlands and [3]Laboratory for Pathology East Netherlands (LabPON), Hengelo, The Netherlands and [4]Department of Pathology, Canisius-Wilhelmina Hospital, Nijmegen, The Netherlands and [5]Department of Pathology, Rijnstate Hospital, Pathology-DNA, Arnhem, The Netherlands

[*]geert.litjens@radboudumc.nl
[†]Contributed equally.

Abstract

Background The presence of lymph node metastases is one of the most important factors in breast cancer prognosis. The most common strategy to assess the regional lymph node status is the sentinel lymph node procedure. The sentinel lymph node is the most likely lymph node to contain metastasized cancer cells and is excised, histopathologically processed and examined by the pathologist. This tedious examination process is time-consuming and can lead to small metastases being missed. However, recent advances in whole-slide imaging and deep learning have opened an avenue for analysis of digitized lymph node sections with computer algorithms. Convolutional neural networks, a type of deep learning algorithm, are able to automatically detect cancer metastases in lymph nodes with high accuracy. To train deep learning models, large, well-curated datasets are needed. **Results** We released a dataset of 1399 annotated whole-slide images of lymph nodes, both with and without metastases, in total three terabytes of data. Slides were collected from five different medical centers to cover a broad range of image appearance and staining variations. Each whole-slide image has a slide-level label indicating whether it contains no metastases, macro-metastases, micro-metastases or isolated tumor cells. Furthermore, for 209 whole-slide images, detailed hand-drawn contours for all metastases are provided. Last, open-source software tools to visualize and interact with the data have been made available. **Conclusions** A unique dataset of annotated, whole-slide digital histopathology images has been provided with high potential for re-use.

Key words: breast cancer; lymph node metastases ; whole-slide images; grand challenge; sentinel node

Background

Breast cancer is one of the most common and deadly cancers in women worldwide [1]. Although prognosis for breast cancer patients is generally good, with an average five-year overall survival rate of 90% and ten-year survival rate of 83%, it significantly deteriorates when breast cancer metastasizes [2]. While localized breast cancer has a five-year survival rate of 99%,

Compiled on: December 18, 2017.
Draft manuscript prepared by the author.

this drops to 85% in the case of regional (lymph node) metastases and only 26% in case of distant metastases. As such, it is of the utmost importance to establish whether metastases are present to allow adequate treatment and the best chance of survival. This is formally captured in the TNM staging criteria [3].

The first step in determining the presence of metastases is the examination of the regional lymph nodes. Not only is the presence of metastases in these lymph nodes a poor prognostic factor by itself, it is also an important predictive factors for the presence of distant metastases [4]. In breast cancer the most common strategy to assess the regional lymph node status is the sentinel lymph node procedure [5, 6]. Within this procedure a blue dye and/or radioactive tracer is injected near the tumor. The lymph node reached first by the injected substance, the sentinel node, is most likely to contain the metastasized cancer cells and is excised. Subsequently, it is submitted for histopathological processing and examination by the pathologist.

Table 1. Rules for assigning clusters of metastasized tumor cells to a metastasis category.

Category	Size
Macro-metastasis	Larger than 2 mm
Micro-metastasis	Larger than 0.2 mm and/or containing more than 200 cells, but not larger than 2 mm
Isolated tumor cells	Single tumor cells or a cluster of tumor cells not larger than 0.2 mm or less than 200 cells

Pathologists examine a glass slide containing a tissue section of the lymph node stained with hematoxylin and eosin (H&E). Based solitary tumor cells or the diameter of clusters of tumor cells, metastases can be divided in one of three categories: macro-metastases, micro-metastases or isolated tumor cells (ITC). The size criteria for each of these categories is shown in Table 1. Based on the presence or absence of one or more of these metastasis an initial pathological N-stage (pN) is assigned to a patient. Based on this initial stage, in combination with characteristics of the main tumor, further lymph node dissection or axillary radiotherapy may be performed. These axillary lymph nodes are then also pathologically assessed to come to a final pN-stage. pN categorization is mostly based on metastasis size and the number of lymph nodes involved, but also on the anatomical location of the lymph nodes. A small excerpt of the pN stage is shown in Table 2; for a full listing we refer to the 7th edition of the TNM staging criteria for breast cancer [7].

A key challenge for pathologists in assessing lymph node status is the large area of tissue that has to be examined to identify metastases that can be as small as single cells. Examples of a macro-metastasis, micro-metastasis, and ITC are shown in Figure 2. For sentinel lymph nodes at least three sections at different levels through the lymph node have to be examined and for non-sentinel lymph nodes one section of at least ten lymph nodes has to be examined [8, 9]. This tedious examination process is time-consuming and pathologists may miss small metastases [10]. In the Netherlands, a secondary examination using an immunohistochemical staining for cytokeratin has to be performed if inspection of the H&E-slide identifies no metastases. However, even in this secondary examination, metastases can still be missed [11].

Nowadays, advances in whole-slide imaging and deep learning have opened an avenue for analysis of digitized lymph nodes sections with computer algorithms. Whole-slide imaging is a technique where high-speed slide scanners digitize glass slides at very high resolution (e.g. 240 nm per pixel). This results in images with a size in the order of 10 gigapixels, typically called whole-slide images (WSI). This large amount of data makes WSIs ideally suited for analysis with deep learning algorithms, like convolutional neural networks (CNNs). We were the first to show that training CNNs to detect cancer metastases in lymph nodes was possible and potentially could result in improved efficiency and accuracy of histopathologic diagnostics [12].

Table 2. Selection of N-stages for staging of breast cancer based on the 7th edition of the TNM-criteria.

Stage	Description
N0	Cancer has not spread to nearby lymph nodes.
N0(i+)	The lymph nodes only contains ITCs
N1mi	Micro-metastases in 1 to 3 lymph nodes axillary
N1a	Cancer has spread to 1 to 3 lymph nodes axillary with at least one macro-metastasis
N1b	Cancer has spread to internal mammary lymph nodes, but this spread could only be found on sentinel lymph node biopsy
N1c	Both N1a and N1b apply
N2a	Cancer has spread to 4 to 9 lymph nodes under the arm, with at least one macro-metastasis
N2b	Metastases in clinically detected internal mammary lymph nodes in the absence of axillary lymph node metastases

To train deep learning models, large, well-curated datasets are needed to both train these models and accurately evaluate their performance. To allow the broader computer vision community to replicate and build on our results, we publicly released a large dataset of annotated whole-slide images of lymph nodes, both with and without metastases in the context of the CAMELYON16 and CAMELYON17 challenges (CAncer MEtastases in LYmph nOdes challeNge) [13, 14]. This dataset was collected at different Dutch medical centers to cover the heterogeneity encountered in clinical practice. It contains a total of 1399 WSIs, resulting in approximately three terabytes of image data.

Within these challenges, we released a part of the dataset with the reference standard (i.e. the training set) to allow other groups to build algorithms to detect metastases. Subsequently, the rest of the dataset was released without reference standard (i.e. the test set). Participating teams could submit their algorithm output on the test set to us, after which we evaluated their performance on a predefined set of metrics to allow fair and standardized comparison to other teams. To enable participation of teams that are not familiar with whole-slide images, we released a publicly available software package for viewing WSIs, annotations and algorithmic results, dubbed the Automated Slide Analysis Platform (ASAP) [15].

This paper describes the CAMELYON dataset in detail, and covers the following topics:

- Sample collection
- Slide digitization and conversion
- Challenge dataset construction and statistics
- Instructions on the use of ASAP to view and analyze slides
- Suggestions for data re-use

Data Description

The CAMELYON dataset is a combination of the WSIs of sentinel lymph node tissue sections collected for the CAMELYON16 and

CAMELYON17 challenges, which contained 399 WSIs and 1000 WSIs, respectively. This resulted in a total of 1399 unique WSIs and a total data size of 2.95 terabytes. The dataset is currently publicly available after registration via the CAMELYON17 website [14]. At the time of writing it has been accessed by over 800 registered users worldwide. It has been licensed under the Creative Commons CC BY-NC-ND 4.0 license.

Table 3. WSI-level characteristics for the CAMELYON16 part of the dataset.

Center	Total WSIs	Metastases		
		None	Macro	Micro
RUMC	249	150	49	50
UMCU	150	90	34	26

Table 4. WSI-level characteristics for the CAMELYON17 part of the dataset.

Center	Total WSIs		Metastases (Train)			
	Train	Test	None	Macro	Micro	ITC
CWZ	100	100	61	15	11	13
LPON	100	100	59	26	7	8
RST	100	100	58	12	24	6
RUMC	100	100	60	20	14	6
UMCU	100	100	75	15	8	2
Total	500	500	313	88	64	35

Table 5. Patient-level characteristics for the CAMELYON17 part of the dataset.

Center	Total patients		Stages (Train)				
	Train	Test	pN0	$pN0_{i+}$	$pN1_{mi}$	pN1	pN2
CWZ	20	20	4	3	5	6	2
LPON	20	20	5	2	3	5	5
RST	20	20	4	2	5	5	4
RUMC	20	20	3	3	3	6	5
UMCU	20	20	8	2	4	3	3
Total	100	100	24	12	20	25	19

Data collection

Collection of the data was approved by the local ethical committee of the Radboud University Medical Center (RUMC) under 2016-2761 and the need for informed consent was waived. Data was collected at five different medical centers in the Netherlands: the RUMC, the Utrecht University Medical Center (UMCU), the Rijnstate Hospital (RST), the Canisius-Wilhelmina Hospital (CWZ), and LabPON (LPON). An example of digitized slides from these centers can be seen in Figure 1.

Initial identification of cases eligible for inclusion was based on local pathology reports of sentinel lymph node procedures between 2006 and 2016. The exact years included varied from center to center, but did not affect data distribution or quality. After the lists of sentinel node procedures and the corresponding glass slides containing H&E-stained tissue sections were obtained, slides were randomly selected for inclusion. As the vast majority of sentinel lymph nodes are negative for metastases, selection was stratified for the presence of macro-metastases, micro-metastases and ITCs based on the original pathology reports. This was done to obtain a good representation of differing metastasis appearance without the need for an excessively large dataset.

Data was acquired in two stages, corresponding to the time periods for organization of the CAMELYON16 and CAMELYON17-challenge. Within the CAMELYON16 challenge, only data from the RUMC and UMCU was acquired and no slides containing only ITCs were included. For CAMELYON17 data was included from all five centers and glass slides containing only ITCs were obtained as well. A categorization of the slides can be found in Tables 3 and 4.

After selection of the glass slides, they were digitized with different slide scanners such that scan variability across centers was captured in addition to H&E-staining procedure variability. The slides from RUMC, CWZ and RST were scanned with the 3DHistech Pannoramic Flash II 250 scanner at the RUMC. At the UMCU slides were scanned with a Hamamatsu NanoZoomer-XR C12000-01 scanner and at LPON with a Philips Ultrafast Scanner.

As all slides are initially stored in an original vendor format which makes re-use challenging, slides were converted to a common, generic TIFF (Tagged Image File Format) using an open-source file converter, part of the ASAP package [15]. As there are no open-source tools to convert the iSyntax format produces by the Philips Ultrafast Scanner a proprietary converter was used to convert files to a special TIFF format[16], which can be read by the open-source package OpenSlide [17] and the ASAP package [15].

After digitization, the reference standard for each slide needed to be established. The reference standard for each WSI consisted of a slide level label indicating the largest metastasis within a slide (i.e. no metastasis, macro-metastasis, micro-metastasis or ITC). Furthermore, for all 399 WSIs which were part of the CAMELYON16 challenge and an additional 50 WSIs from the CAMELYON17-challenge detailed contours were drawn along the boundaries of metastases within the WSI. For the 50 slides of the CAMELYON17 challenge, 10 slides from each center were used to allow users of the dataset to analyze metastasis appearance differences across different centers.

Initial slide level labels were assigned based on the pathology reports obtained from clinical routine. For the CAMELYON16 part of the dataset all slides were subsequently examined and metastases outlined by an experienced lab technician (M.H.) and a clinical PhD student (Q.M.). Afterwards, all annotations were inspected by one of two expert breast pathologists (P.B. or P.v.D.). Some slides contained two consecutive tissue sections of the same lymph node, in which case only one of the two sections was annotated as this did not affect the slide level label. In total 15 slides may contain unlabeled metastatic areas and are indicated via a descriptive text file which is part of the dataset.

For the CAMELYON17 part of the dataset an experienced general pathologist (M.v.D.) inspected all the slides to assess the slide level labels. For the 50 slides with detailed annotations, experienced observers (M.v.D., M.H., Q.M., O.G. and R.vd.L.) annotated all metastases. Subsequently, these annotations were double-checked by one of the other observers or one of two pathology residents (A.H. and R.V.).

For the entire dataset, when the slide level label was unclear during the inspection of the H&E-stained slide, an additional WSI with a consecutive tissue section, immunohistochemically (IHC) stained for cytokeratin, was used to confirm the classification. This stain can also be used in daily clinical pathology practice to resolve diagnosis in the case of metastasis-negative H&E [18, 19]. An example of an H&E WSI and the corresponding consecutive cytokerain immunohistochemical section is shown in Figure 3.

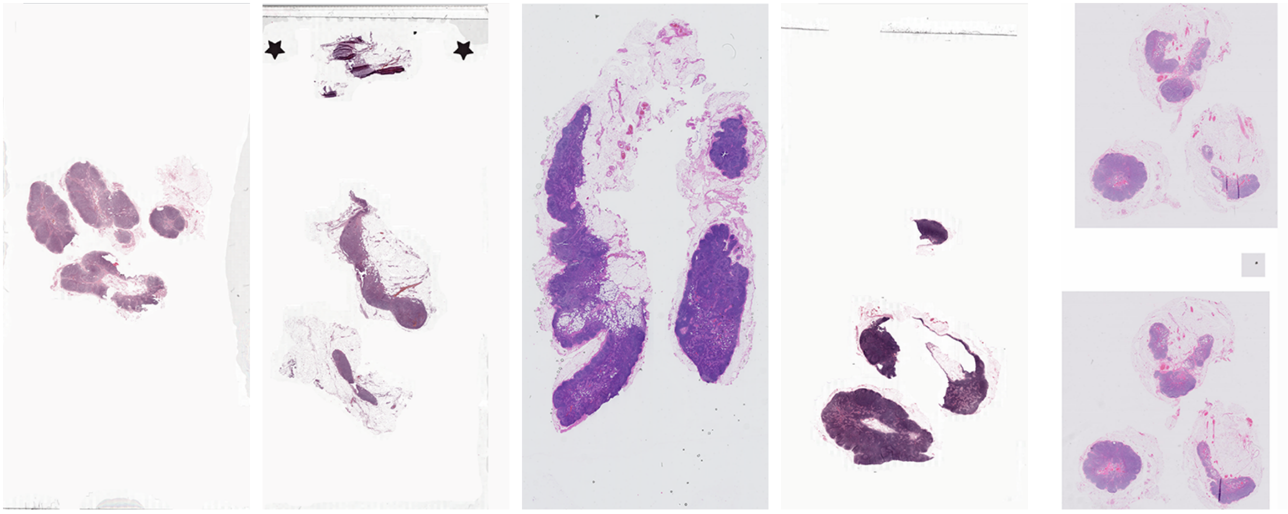

Figure 1. Low-resolution example of a whole-slide image from each of the five centers contributing data.

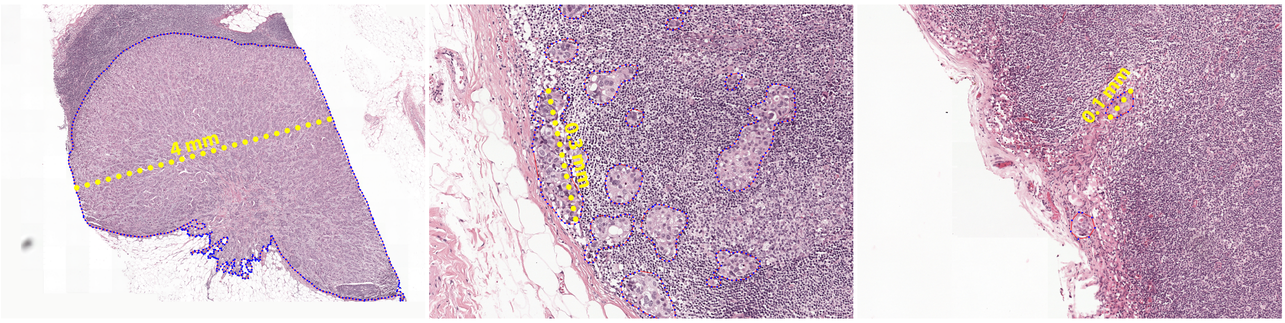

(a) Macro-metastasis **(b)** Micro-metastasis **(c)** Isolated tumor cells

Figure 2. Representative samples of the different sizes of breast cancer metastases in sentinel lymph nodes.

In the CAMELYON17 dataset, after establishing the reference standard, slides were divided into artificial patients, covering the different pN-stages (see Table 2). Each artificial patient only had WSIs from one center. For each artificial patient in the training part of the dataset the pN-stage and the slide level labels were provided. This was done to assess the potential of participating algorithms within the challenge to perform automated pN-staging. However, all WSIs can be used independently of their patient level labels.

After the dataset and reference standard were established we uploaded the entire dataset to Google Drive and to BaiduPan. These two options were chosen to reach as wide an audience as possible, given that Google Drive is not accessible everywhere (e.g. People's Republic of China). A link to the data was shared with participants after registration at the CAMELYON-websites [13, 14].

Data validation and quality control

All slides included in the CAMELYON-dataset were part of routine clinical care and are thus of diagnostic quality. After scanning, all slide were manually inspected by an experienced technician (Q.M and N.S. for center UMCU, M.H. or R.vd.L. for the other centers) to assess scan quality and when in doubt a pathologist was consulted.

Due to the inclusion of IHC for establishing the reference standard the chance of errors being made can be considered limited, as pathologists make few mistakes in identifying metastases with IHC [20]. Furthermore, all slides were checked twice. However, to further ensure the quality of the reference standard we looked at algorithmic results submitted to the challenge to identify slides where the best performing algorithms disagreed with the reference standard. This led to a correction of the reference standard in 3 of the 1399 slides.

Tools for data use

Several tools are available to visualize and interact with the CAMELYON-dataset. Here we will present examples of how to use the data with an open-source package developed by us, called ASAP (Automated Slide Analysis Platform) [15]. Other open-source packages are also available, such as OpenSlide [21], but those do not contain functionality for reading annotations or storing image analysis results.

- Project name: Automated Slide Analysis Platform (ASAP)
- Project home page: https://github.com/GeertLitjens/ASAP
- Operating system(s): Linux, Windows
- Programming language: C++, Python
- Other requirements: CMake (www.cmake.org)
- License: GNU GPL v2.0

ASAP contains several components, of which one is a viewer/annotation application (Figure 4). This can be started via the ASAP executable within the installation folder of the package. After opening an image file from the CAMELYON-dataset one can explore the data via a 'Google Maps'-like interface. The provided reference standard can be loaded via the annotation plugin. Furthermore, new annotations can be made with the provided annotation tools. Last, the viewer is not lim-

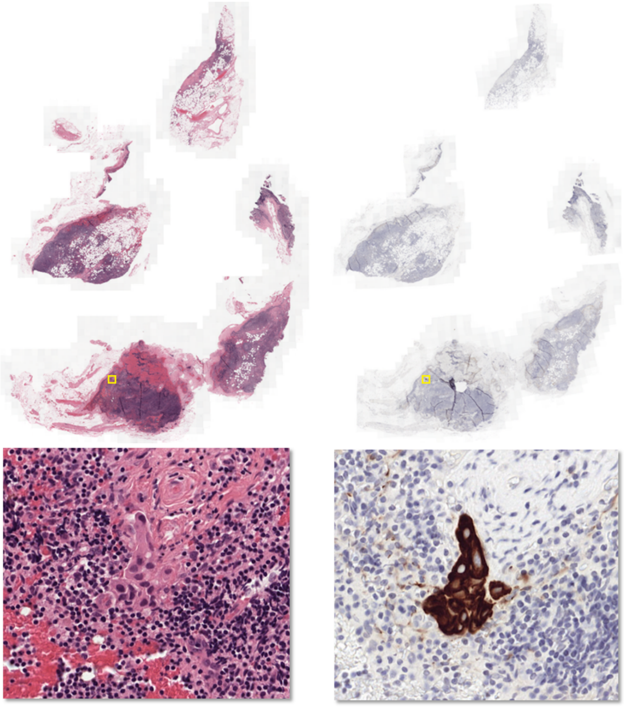

Figure 3. H&E-stained tissue section and a consecutive section immunohistochemically stained for cytokeratin. The top row shows the low-resolution images and the bottom row a high-resolution image, centered at a metastasis. The metastasis is difficult to see in H&E, but easy to identify in the immunohistochemically-stained slide. A yellow bounding box indicates the metastasis location in the images in the top row.

ited to files from CAMELYON-dataset but can visualize most WSI formats.

In addition to the viewer application and C++ library to read and write WSI images, we also provide Python-wrapped modules. To access the data via Python the following code-snippet can be used.

```
# Example of extracting and visualizing
# image data from the CAMELYON-dataset.
import multiresolutionimageinterface as mir
import matplotlib.pyplot as plt

reader = mir.MultiResolutionImageReader()
image = reader.open("Normal_001.tif")
# "Normal_001.tif" should be replaced
# with the path to that specific file.

# Gets the complete image at resolution
# level 6 (low resolution) and plot it.
dims = image.getLevelDimensions(6)
tile = image.getUCharPatch(0, 0, dims[0], dims[1], 6)
plt.imshow(tile)

# Get a high resolution tile of the image
# at level 0 and plot it
tile = image.getUCharPatch(37000, 90000, 1024, 1024, 0)
plt.imshow(tile)
```

The annotations are provided in human-readable XML format and can be parsed using the ASAP-package. However, other XML reading libraries can also be used. Annotations are stored as polygons. Each polygon consists of a list of (x, y) coordinates at the highest resolution level of the image. Annotations can be converted to binary images via the following code-snippet.

```
# Example of converting an annotation to
# an indexed mask image.

# Reads a specific image from the CAMELYON17 dataset
import multiresolutionimageinterface as mir
reader = mir.MultiResolutionImageReader()
image = reader.open('patient_010_node_4.tif')

# Loads the list of annotations from disk
annotation_list = mir.AnnotationList()
xml_repository = mir.XmlRepository(annotation_list)
xml_repository.setSource('patient_010_node_4.xml')
xml_repository.load()

# Access the first annotation (index 0)
# and print the area, number of points and
# x-coordinate of the first point.
annotation = annotation_list.getAnnotation(0)
print(annotation.getArea())
print(annotation.getNumberOfPoints())
print(annotation.getCoordinate(0).getX())

# Convert the annotations to an indexed image
annotation_mask = mir.AnnotationToMask()
label_map = {'metastases': 1, 'normal': 2}
output_path = 'patient_010_node_4_labels.tif'
annotation_mask.convert(annotation_list, output_path,
                        image.getDimensions(),
                        image.getSpacing(), label_map)
```

The Python package can also be used to perform image processing or machine learning tasks on the data and write out an image result. The code-snippet below performs some basic thresholding to generate a background mask. These results can then subsequently be visualized using the viewer component of ASAP, which also supports floating point images. An example of the code-snippet result can be seen in Figure 4b.

```
import multiresolutionimageinterface as mir
import numpy as np
from scipy.ndimage.filters import median_filter
from skimage.transform import resize

reader = mir.MultiResolutionImageReader()
image = reader.open("Normal_001.tif")
level_dims = image.getLevelDimensions(6)
level_ds = image.getLevelDownsample(6)
tile = image.getUCharPatch(0, 0, level_dims[0],
                           level_dims[1], 6)
tile_clipped = np.clip(tile, 1, 254)
tile_od = -np.log(tile_clipped / 255.)
D = median_filter(np.sum(tile_od, axis=2) / 3., size=3)
raw_mask = (((D > 0.03 * -np.log(1/255.)) *
            (D < 0.97 * -np.log(1/255.))
           ).astype("ubyte"))

out_dims = image.getLevelDimensions(0)
step_size = int(512. / int(level_ds))
writer = mir.MultiResolutionImageWriter()
writer.openFile("Normal_001_mask.tif")
writer.setTileSize(512)
writer.setCompression(mir.LZW)
writer.setDataType(mir.UChar)
writer.setInterpolation(mir.NearestNeighbor)
writer.setColorType(mir.Monochrome)
```

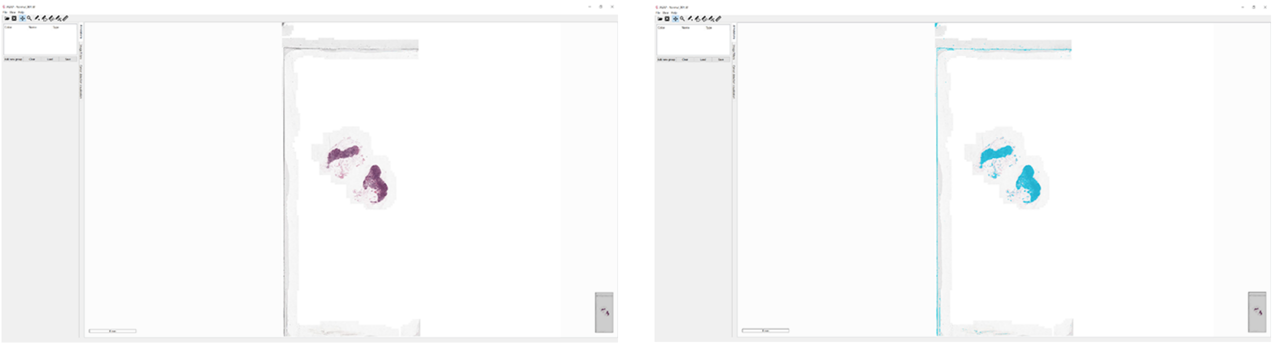

(a) Without tissue mask overlay

(b) With tissue mask overlay in blue

Figure 4. Interface of the Automated Slide Analysis Platform (ASAP) viewer interface. Visible items are the annotations tools in toolbar, the viewport showing the WSI and the plugin panel on the left.

```
writer.writeImageInformation(out_dims[0], out_dims[1])
for y in range(0, level_dims[1], step_size):
  for x in range(0, level_dims[0], step_size):
        write_tl = np.zeros((step_size, step_size),
                        dtype='ubyte')
        cur_tl = raw_mask[y:y+step_size,
                      x:x+step_size]
        write_tl[0:cur_tl.shape[0],
             0:cur_tl.shape[1]] = cur_tl
        res_tl = resize(write_tl, (512,512), order=0,
                    mode="constant",
                    preserve_range=True).astype("ubyte")
        writer.writeBaseImagePart(res_tl.flatten())
writer.finishImage()
```

The ASAP package also supports writing your own image processing routines and integrating them as plugins into the viewer component. Some existing examples like color deconvolution and nuclei detection are provided.

Re-use potential

The CAMELYON dataset is currently still being used within the CAMELYON17 challenge, which is open for new participants and submissions. In this context, the dataset enables testing new machine learning and image analysis strategies against the current state-of-the-art. Conclusions arising from such experiments may have significance for the broader field of computational pathology, rather than being restricted to this particular application. For example, experiments with weakly supervised machine learning in histopathology may benefit from the CAMELYON dataset, with an established baseline based on fully supervised machine learning.

The dataset has also been used by companies experienced in machine learning application to be a first foray into digital pathology, for example Google [22]. Because of its extent, observer experiments with pathologists may be performed to assess the value of algorithms within a diagnostic setting. For example, a comparison of algorithms competing in the CAMELYON16-challenge to pathologists in clinical practice was recently published [23]. Experiments with the dataset may serve to identify relevant issues with implementation, validation and regulatory affairs with respect to computational pathology.

We believe the usefulness of the dataset also extends beyond it's initial use within the CAMELYON-challenge. For example, it can be used for evaluation of color normalization algorithms, and for cell detection/segmentation algorithms.

Declarations

List of abbreviations

ASAP Automated Slide Analysis Platform
H&E Hematoxylin and eosin
IHC Immunohistochemistry
ITC Isolated tumor cells
WSI Whole-slide image

Ethical Approval

Collection of the data was approved by the local ethical committee ('Commissie Mensgebonden Onderzoek regio Arnhem – Nijmegen') under 2016-2761 and the need for informed consent was waived.

Competing Interests

Jeroen van der Laak, Paul van Diest and Alexi Baidoshvilli are members of the scientific advisory board of Philips Digital Pathology (Best, The Netherlands). Jeroen van der Laak is also part of the scientific advisory board of ContextVision (Stockholm, Sweden), and Paul van Diest of the scientific advisory board of Sectra (Linköping, Sweden).

Funding

Data collection and annotation where funded by Stichting IT Projecten and by the Fonds Economische Structuurversterking (tEPIS/TRAIT project; LSH-FES Program 2009; DFES1029161 and FES1103JJTBU). This work was also supported by grant 601040 from the FP7-funded VPH-PRISM project of the European Union.

Author's Contributions

GL and JvdL designed the study and supervised the collection of the dataset; GL wrote the initial draft and final version of the paper; PBu, OG, BEB, MB, MH, QM, AB, NS, PvD, MvD and CW were involved in sample collection; GL, PBa and NS were involved in data anonymization and conversion; PBu, OG, MH, MB, MvD, QM, AH, RV, PvD were involved in establishing the reference standard. All authors were involved in reviewing and finalizing the paper.

References

1. Siegel RL, Miller KD, Jemal A. Cancer statistics, 2016. CA Cancer J Clin 2016;66(1):7–30.
2. Howlader N, Noone AM, Krapcho M, Miller D, Bishop K, Kosary CL, et al. SEER Cancer Statistics Review, 1975-2014, National Cancer Institute. Bethesda, MD, http://seer.cancer.gov/csr/1975_2014/ based on November 2016 SEER data submission, posted to the SEER web site, April 2017;http://seer.cancer.gov/csr/1975_2014/.
3. Amin MB, Edge SB, Greene FL, Byrd DR, Brookland RK, Washington MK, et al. AJCC Cancer Staging Manual. Springer-Verlag GmbH; 2016. http://www.ebook.de/de/product/26196032/ajcc_cancer_staging_manual.html.
4. Voogd AC, Nielsen M, Peterse JL, Blichert-Toft M, Bartelink H, Overgaard M, et al. Differences in risk factors for local and distant recurrence after breast-conserving therapy or mastectomy for stage I and II breast cancer: pooled results of two large European randomized trials. Journal of clinical oncology : official journal of the American Society of Clinical Oncology 2001 Mar;19:1688–1697.
5. Giuliano AE, Hunt KK, Ballman KV, Beitsch PD, Whitworth PW, Blumencranz PW, et al. Axillary dissection vs no axillary dissection in women with invasive breast cancer and sentinel node metastasis: a randomized clinical trial. JAMA 2011 Feb;305:569–575.
6. Giuliano AE, Ballman KV, McCall L, Beitsch PD, Brennan MB, Kelemen PR, et al. Effect of Axillary Dissection vs No Axillary Dissection on 10-Year Overall Survival Among Women With Invasive Breast Cancer and Sentinel Node Metastasis: The ACOSOG Z0011 (Alliance) Randomized Clinical Trial. JAMA 2017 Sep;318:918–926.
7. Edge SB, Compton CC. The American Joint Committee on Cancer: the 7th edition of the AJCC cancer staging manual and the future of TNM. Ann Surg Oncol 2010 Jun;17:1471–1474.
8. Weaver DL. Pathology evaluation of sentinel lymph nodes in breast cancer: protocol recommendations and rationale. Mod Pathol 2010;23 Suppl 2:S26–S32.
9. Somner JEA, Dixon JMJ, Thomas JSJ. Node retrieval in axillary lymph node dissections: recommendations for minimum numbers to be confident about node negative status. Journal of clinical pathology 2004 Aug;57:845–848.
10. van Diest PJ, van Deurzen CHM, Cserni G. Pathology issues related to SN procedures and increased detection of micrometastases and isolated tumor cells. Breast disease 2010;31:65–81.
11. Vestjens J, Pepels M, de Boer M, Borm GF, van Deurzen CH, van Diest PJ, et al. Relevant impact of central pathology review on nodal classification in individual breast cancer patients. Ann Oncol 2012;23(10):2561–2566.
12. Litjens G, Sánchez CI, Timofeeva N, Hermsen M, Nagtegaal I, Kovacs I, et al. Deep learning as a tool for increased accuracy and efficiency of histopathological diagnosis. Nat Sci Rep 2016;6:26286. http://dx.doi.org/10.1038/srep26286.
13. The CAMELYON16 Challenge; 2017. Accessed: 2017-11-13. https://camelyon16.grand-challenge.org.
14. The CAMELYON17 Challenge; 2017. Accessed: 2017-11-13. https://camelyon17.grand-challenge.org.
15. Litjens GJS, Automate Slide Analysis Platform (ASAP); 2017. Accessed: 2017-10-17. https://github.com/geertlitjens/ASAP.
16. Description of Philips TIFF file format; 2017. Accessed: 2017-10-17. http://openslide.org/formats/philips/.
17. Goode A, Gilbert B, Harkes J, Jukic D, Satyanarayanan M, et al. OpenSlide: A vendor-neutral software foundation for digital pathology. Journal of pathology informatics 2013;4(1):27.
18. Chagpar A, Middleton LP, Sahin AA, Meric-Bernstam F, Kuerer HM, Feig BW, et al. Clinical outcome of patients with lymph node-negative breast carcinoma who have sentinel lymph node micrometastases detected by immunohistochemistry. Cancer 2005;103:1581–1586.
19. Reed J, Rosman M, Verbanac KM, Mannie A, Cheng Z, Tafra L. Prognostic implications of isolated tumor cells and micrometastases in sentinel nodes of patients with invasive breast cancer: 10-year analysis of patients enrolled in the prospective East Carolina University/Anne Arundel Medical Center Sentinel Node Multicenter Study. J Am Coll Surg 2009;208:333–340.
20. Roberts CA, Beitsch PD, Litz CE, Hilton DS, Ewing GE, Clifford E, et al. Interpretive disparity among pathologists in breast sentinel lymph node evaluation. Am J Surg 2003 Oct;186(4):324–329.
21. OpenSlide; 2017. Accessed: 2017-10-17. http://openslide.org.
22. Liu K, Kang G. Multiview convolutional neural networks for lung nodule classification. International Journal of Imaging Systems and Technology 2017;27(1):12–22.
23. Ehteshami Bejnordi B, Veta M, van Diest PJ, van Ginneken B, Karssemeijer N, Litjens G, et al. Diagnostic Assessment of Deep Learning Algorithms for Detection of Lymph Node Metastases in Women With Breast Cancer. JAMA 2017 Dec;318:2199–2210.
