# Supplementary material for: 1399 H&E-stained sentinel lymph node sections of breast cancer patients: the CAMELYON dataset
Source: Gigascience. 2018 May 31;7(6):giy065. doi: 10.1093/gigascience/giy065 (PMC6007545; doi:10.1093/gigascience/giy065)
Supplement: Supplement Files [file giy065_supplement_files.zip › gigascience-logo.pdf]

(GIGA)^n SCIENCE
