# Supplementary figures and images for: 1399 H&E-stained sentinel lymph node sections of breast cancer patients: the CAMELYON dataset
Source: Gigascience. 2018 May 31;7(6):giy065. doi: 10.1093/gigascience/giy065 (PMC6007545; doi:10.1093/gigascience/giy065)

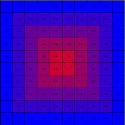

Supplement: Supplement Files [file giy065_supplement_files.zip › example-grid-100x100pt.png]

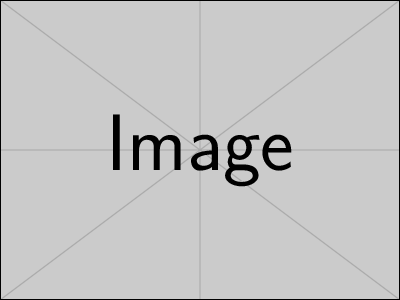

Supplement: Supplement Files [file giy065_supplement_files.zip › example-image.png]
