# Supplementary material for: 1399 H&E-stained sentinel lymph node sections of breast cancer patients: the CAMELYON dataset
Source: Gigascience. 2018 May 31;7(6):giy065. doi: 10.1093/gigascience/giy065 (PMC6007545; doi:10.1093/gigascience/giy065)
Supplement: Response_to_Reviewer_Comments_Original_Submission.pdf [file giy065_response_to_reviewer_comments_original_submission.pdf]

Author's Response To Reviewer Comments

Close

First, we would like to thank both reviewers for their insightful comments. We have done our best to address all comments adequately and feel the paper has improved as a result. We provide detailed responses to each of the individual comments below. We have also color-coded all changes in the revised paper with a red font so reviewers can easily identify changes.

Reviewer #1, question 1:
The manuscript describes a dataset of H&E stained slides for breast cancer pathology, and is made available for the primary purpose of computer-based diagnostics and prognosis of breast cancer. Open datasets and benchmarks are very important tools with proven success in advancing different fields, especially related to pattern recognition, and it is likely that a clean and open dataset will be used by many, as the dataset is already being used and already making impact. The paper itself is a short well-written piece that describes the work well, and can be used as a base reference to this project. This reviewer believes that the work is useful and justifies publication, but would like to make several suggestions before the work is published. I made all efforts to give submit this report in a timely manner, and will be quick to respond should further discussion is required.

Answer 1:
We are happy the reviewer agrees with our assessment that the CAMELYON dataset can be a highly useful benchmark for pattern recognition and machine learning techniques. We have addressed all the comments provided by the reviewer below.

Question 2:
For some reason the paper, and especially the abstract, gives the impression that the dataset was created specifically for deep learning. I suggest to make it more general for computer-based diagnostics, as the data itself has very little to do with deep learning, and in fact any method can be tested using these data. Such methods can include also automatic model-driven methods that mimic the work of the pathologist, rather than the data-driven deep learning and other related approaches. Deep learning might be a "buzzword" in 2018, but five years from now there might be another buzzword, but the data will probably still be useful and relevant (H&E has been used for many years). Similar statements are also made in the Background section: "To train deep learning models, large, well-curated datasets are needed to both train these models and accurately evaluate their performance". The sentence is logically correct, but such data are required for training any machine learning model, not just deep learning.

Answer 2:
We agree with the reviewer that the usefulness of the dataset is not limited to deep learning algorithms. As such we have generalized the text to focus on machine learning and pattern recognition models in general.

Question 3:
The claim that "deep learning have opened an avenue…" is an overstatement, as algorithms that are not based on deep learning demonstrated good recognition accuracy in pathology, in fact as early as the 1990's, without using deep learning. That whole sentence gives the

impression that automatic classification of H&E slides for pathology is a new field, while it clearly isn't. I therefore recommend to weaken the statement or make it more general to machine learning. It seems to me that the term "deep learning" is confused with the term "machine learning".

Answer 3:
The reviewer is correct to point out that the analysis of H&E images with machine learning and image analysis methods has been around for several decades. We have updated the text to acknowledge this.

Question 4:
Page 3: The image file format is discussed (TIFF), but without important details. What is the resolution of the images? What is the dynamic range? Bits per pixel? Channels per pixel? Data type (integer, floating point)? etc.

Answer 4:
We have added a table describing the details of the file format to the paper:

Format tiled TIFF (bigTIFF)
Tile size in pixels 512
Pixel resolution 0.23 (Hamamatsu), 0.24 (3DHistech) or 0.25 (Philips) um per pixel
Channels per pixel 3 (Red, green, blue)
Bits per channel 8
Data type Unsigned char
Compression JPEG

Question 5:
The data annotation process is not entirely clear. Pathology can be subjective and different pathologists might reach different conclusions regarding the same slide. The important information about the data annotation is a little vague. For instance, what was the disagreement rate between the pathologists in the different stages? In how many of the cases the inspection by the breast cancer pathologist (PB or PvD) led to a change in the label?

Answer 5:
We agree with the reviewer that there could be variability between pathologists in assessing H&E slides. However, when constructing the reference standard for CAMELYON, in case of uncertainty, the additional immunohistochemistry stain was always available. As indicated in the paper with reference 23, the observer variability in this stain is limited. We have added the following sentence to the paper to further clarify the annotations:

Furthermore, this stain was also used to aid in drawing the outlines in both CAMELYON16 and CAMELYON17, which helps limit observer-variability. As both the H&E and IHC slides are digital, they can be viewed simultaneously, allowing observers to easily identify the same areas in both slides.

Sadly, during the construction of the dataset we did not monitor how often a correction was made by the experienced pathologists. After consulting with them they indicate that this was very rare. To give some number on the strength of the reference standard and potential observer variability, we can give two examples: Google hired a pathologist to check the CAMELYON16 dataset to assess false-positives they had in the challenge. This led to a

correction of the reference standard in only 2 out of 399 cases. For CAMELYON17 we had the slides rechecked again by another pathology resident after receiving the reviews. The resident had access to all immunohistochemically-stained slides as well which led to a correction of 2 slides out of the 1000. So in total 4 slides were relabeled out of 1399 after subsequent extra inspections (< 0.3%), which we think shows that there is limited variability within the reference standard.

Question 6:
I have some painful experience with benchmark dataset that did not really reflect just the real-world problem they were collected for.
http://onlinelibrary.wiley.com/doi/10.1111/j.1365-2818.2011.03502.x/abstract
https://link.springer.com/article/10.1007%2Fs11263-008-0143-7
http://ieeexplore.ieee.org/document/7299607/?reload=true&arnumber=7299607
These can be most risky when using deep learning, where the features are not intuitive and the only practical way to validate the reliability of the results is careful design of the dataset and sound controls.
Apparently, such algorithms can identify the imaging device, and in some cases even the technician acquiring the images, sometimes leading to good prediction accuracy achieved without solving the original problem (as shown in the links above). Therefore, it is not uncommon that models show good accuracy when using the same dataset separated to training and test data, but much lower accuracy when tested with data from a different set. That can even happen with images collected from the internet:
http://ieeexplore.ieee.org/document/5995347/ The dataset described in this paper combines data from multiple medical centers and using different imaging devices, which is good. However, the dataset is still based on a fixed number of sources, and therefore algorithms showing good performance might still be limited to the specific data used in the dataset, and there is no guarantee that the same algorithm performs well also on data from sources it had not "seen" and trained with. As I proposed in the past, one way of solving a problem of this kind is to use data acquired from one center for training, and data from a different center for testing. Good results achieved using this experimental design indicate that the algorithm is not limited to a certain dataset. From the paper it seems that data from all centers were used for both training and testing, and therefore the current design does not test whether a model trained with the dataset can also annotate data coming from other centers that are not included in the dataset. I understand that after the grand challenge has already started and teams have already submitted their results it will be difficult to make a change in the design. However, a clear discussion about that limitation should be added. My understanding is that even with the current data, if researchers are aware of the issue they can separate the data into different centers and perform such experiment, testing how their algorithm performs on data from a center not used for training data.

Answer 6:
The reviewer is indeed completely right, we also have experience with algorithms learning unexpected things (like recognizing a software version of a scanner) when using a non-representative dataset. We hope to have mitigated that in CAMELYON17 by including data from five different centers with different scanners and staining protocols. We have added a section to the discussion covering this topic. We also indicate there that authors can conduct robustness experiments themselves as they know which center the training slides are from (and can thus omit one). The following text was added:

A key example of implementation issues with respect to machine learning algorithms in

medical imaging is generalization to different centers. In pathology centers can differ in tissue preparation, staining protocol and scanning equipment which each can have a profound impact on image appearance. In the CAMELYON dataset we included data from five centers and three different scanners. We are confident algorithms trained with this data will generalize well. Users of the dataset can even explicitly evaluate this as we have indicated for each image from which center it was obtained. By leaving out one center and evaluating performance on that center specifically the participants can assess the robustness of their algorithms.

Question 7:
The dataset is organized in the form of a grand challenge (like Kaggle, for instance), in which the authors do not release the annotation of the test data, but serve as the judges for teams that submit their results. The evaluation is done on the backend, and without the participation of the teams. The scientific motivation behind that practice should be discussed and explained. Kaggle is a very good service, and the practice of a competition is common in pattern recognition (e.g., ImageNet), but in the context of cancer diagnostics the impact and optimization of scientific return through the form of a grand challenge should be explained. The fact that it is a grand challenge should also be mentioned in the abstract.

Answer 7:
We have addressed this comment within the abstract and in the introduction with the following text:

The concept of challenges in medical imaging and computer vision has been around for nearly a decade. In medical imaging it mostly started with the liver segmentation challenge at the annual MICCAI conference in 2007 and in computer vision the ImageNet Challenge is most widely known. The main goal of challenges, both in medical imaging and in computer vision, is to allow a meaningful comparison of algorithms. In scientific literature, this was often not the case as authors present results on their own, often proprietary, datasets with their own choice of evaluation metrics. In medical imaging this was specifically a problem as sharing medical data is often difficult. Challenges change this by making available datasets and enforcing standardized evaluation. Furthermore, challenges have the added benefit of opening up meaningful research questions to a large community who normally might not have access to the necessary datasets.

Question 8
In the context of that grand challenge, I was looking to find some description of how the results are evaluated, but did not find any information. There is indeed some information in the web site, but the information should also be given in the paper.

Answer 8:
We have added this information to the paper in the re-use potential section:

Within CAMELYON we evaluate the algorithms based on a weighted Cohen's kappa at the pN-stage level. This statistics measures the categorical agreement between the algorithm and the reference standard where a value of 0 indicates agreement at the level of chance and 1 is perfect agreement. The quadratic weighting penalizes deviations of more than one category more severely.

Question 9
Page 4, line 52. The paragraph is a repetition of the previous section.

Answer 9
This paragraph specifically focusses on the quality of the scan. Scanning of slides can potentially fail due to dust on the slide or mechanical defects and as such, as a quality control measure, all slides were checked for these issues. We understand that this might have been unclear from this paragraph and have slightly rewritten it. Now it states:

All glass slides included in the CAMELYON-dataset were part of routine clinical care and are thus of diagnostic quality. However, during the acquisition process scanning can fail or result in out-of-focus images. As a quality control measure, all slides were inspected manually after scanning. The inspection was performed by an experienced technician (Q.M and N.S. for center UMCU, M.H. or R.vd.L. for the other centers) to assess the quality of the scan and when in doubt a pathologist was consulted whether scanning issues might affect diagnosis.

Question 10
Page 6: "The dataset has also been used by companies experienced in machine learning application to be a ¬first foray into digital pathology, for example Google [22]." How is reference 22 related to Google?

Answer 10:
We made a mistake with the reference in Latex, we have updated it to refer to the correct paper.

Reviewer #2, question 1:
In this Data Note, the authors describe a large morphological study of digitised lymph node sections that could be used for exploring the ability of machine-learning algorithms to identify metastases on tissue sections. The lymph node specimens were collected from 5 different medical centres and the histopathological status was scored using TNM staging criteria. In the first study (CAMELYON16), a lab technician and a PhD student performed staging and expert pathologists confirmed the annotations. In a second study (CAMELYON17), a general pathologist staged the lymph node specimens, and detailed annotations were validated by one of two pathology residents. In addition, the authors describe the publicly available Automated Slide Analysis Platform (ASAP) software package that can be used to view whole-slide images, annotations and algorithmic results. The manuscript is well-written and I consider the CAMELYON dataset of great interest to the machine-learning community.

Answer 1:
We thank the reviewer for his kind assessment of both the dataset and the paper. We have tried to address his comments below.

Question 2:
The CAMELYON dataset is available under Creative Commons License CC-BY-NC-ND. This implies that the data is free to share for non-commercial use. However, with this current license agreement the CAMELYON dataset may not be used for commercial

purposes. Furthermore, the CC-BY-NC-ND license agreement implies that derivatives from these material, which could include segmentations of the original image data, may not be distributed commercially or non-commercially. This severely impinges on the utility of this dataset for machine-learning. The authors should consider changing the Creative Commons License agreement for the CAMELYON dataset so that re-use is encouraged.

Answer 2:
We agree with the reviewer and have contacted our partners and have agreed on licensing the dataset under CC-0. This is now also correctly reflected in the text.

Question 3:
I would like more detail on how the polygon tool was used to manually delineate metastases. In particular, could the authors provide details of whether the immunohistochemically-labelled slides stained with anti-cytokeratin were used as a guide for annotating the adjacent H&E sections? Alternatively, were the H&E sections labelled directly without first inspecting the cytokeratin-labelled sections?

Answer 3:
The immunohistochemically-stained slides were indeed used to guide annotations, but annotations were directly made on the H&E. Essentially the annotators used a ‘mental registration’ to identify the corresponding areas, which is usually not difficult as sections are adjacent. We have added the following sentence to the Data collection section to clarify this:

Furthermore, this stain was also used to aid in drawing the outlines in both CAMELYON16 and CAMELYON17, which helps limit observer-variability. As both the H&E and IHC slides are digital, they can be viewed simultaneously, allowing observers to easily identify the same areas in both slides.

Question 4:
In addition, it would be good to know whether a consensus was reached between multiple pathologists in validating the hand-drawn annotations as this may impact on the ability of machine-learning algorithms to computationally identify metastases. Was there a consensus between multiple pathologists for all 399 hand-drawn contours produced from the CAMELYON16 dataset? Similarly, was there a consensus between multiple pathologists for all 50 hand-drawn contours that were produced from the CAMELYON17 dataset?

Answer 4:
No, we did not obtain consensus annotations from multiple pathologists as this would be prohibitively costly in terms of time and available pathologists, given the size of the dataset. However, annotations were guided by immunohistochemically-stained slides and we know there is limited observer variability in those cases. Furthermore, all slides were double-checked by a pathologist or pathology resident with significant experience to prevent any accidental mistakes.

To give some number on the strength of the reference standard and potential observer variability, we can give two examples: Google hired a pathologist to check the CAMELYON16 dataset to assess false-positives they had in the challenge. This led to a correction of the reference standard in only 2 out of 399 cases1. For CAMELYON17 we had the slides rechecked again by another pathology resident after receiving the GigaScience

reviews. The resident had access to all immunohistochemically-stained slides as well which led to a correction of 2 slides out of the 1000. So in total 4 slides were relabeled out of 1399 after subsequent extra inspections (< 0.3%), which we think shows that there is limited variability within the reference standard.

Question 5:
Details of the primary and secondary antibodies used to stain for pan-cytokeratin have not been provided. If the various different medical centres used different antibodies, then this should be clearly stated in the manuscript as it may impact on the ability of machine-learning algorithms to process the immunohistochemically-labelled image data.

Answer 5:
We have collected the information on the antibodies, which we have attached here. However, as the immunohistochemical slides are not part of the CAMELYON dataset, but were only used for the reference standard, we have not added this information to the paper. However, if the reviewer feels this is still valuable we would be happy to add it.

Center Vendor Antibody
CWZ Agilent CK8/18
LabPON Agilent CK8/18
Rijnstate Novacastra CK8/18
Radboud BD Biosciences CAM5.2
UMCU BD Biosciences CAM5.2

Question 6:
Figure 4 shows the tissue mask overlay at low-resolution and it is very difficult to see how accurate the mask overlays the lymph node tissue. The authors should consider revising this figure to include higher-resolution images so that the mask overlay is clearly seen.

Answer 6:
We have added a higher resolution image. However, please note that the goal of that example is not to provide a very good tissue segmentation, but to show that only in a few lines of code a coarse segmentation can easily be created thanks to the library and visualized in the provided viewer.

Close
