# Supplementary material for: 1399 H&E-stained sentinel lymph node sections of breast cancer patients: the CAMELYON dataset
Source: Gigascience. 2018 May 31;7(6):giy065. doi: 10.1093/gigascience/giy065 (PMC6007545; doi:10.1093/gigascience/giy065)
Supplement: Response_to_Reviewer_Comments_Revision1.pdf [file giy065_response_to_reviewer_comments_revision1.pdf]

Author's Response To Reviewer Comments

Close

We thank the reviewers for their positive comments. We have addressed the comment of the editor and added the proposed section.

Close
