# Supplementary material for: 1399 H&E-stained sentinel lymph node sections of breast cancer patients: the CAMELYON dataset
Source: Gigascience. 2018 May 31;7(6):giy065. doi: 10.1093/gigascience/giy065 (PMC6007545; doi:10.1093/gigascience/giy065)
Supplement: Reviewer_1_Report_(Original_Submission) -- Lior Shamir — 1/3/2018 Reviewed [file giy065_reviewer_1_report_(original_submission).pdf]

Reviewer Report

Title: **1399 H&E-stained sentinel lymph node sections of breast cancer patients: the CAMELYON dataset**

Version: **Original Submission** **Date:** 1/3/2018

Reviewer name: **Lior Shamir**

Reviewer Comments to Author:

The manuscript describes a dataset of H&E stained slides for breast cancer pathology, and is made available for the primary purpose of computer-based diagnostics and prognosis of breast cancer.Open datasets and benchmarks are very important tools with proven success in advancing different fields, especially related to pattern recognition, and it is likely that a clean and open dataset will be used by many, as the dataset is already being used and already making impact. The paper itself is a short well-written piece that describes the work well, and can be used as a base reference to this project.This reviewer believes that the work is useful and justifies publication, but would like to make several suggestions before the work is published. I made all efforts to give submit this report in a timely manner, and will be quick to respond should further discussion is required.For some reason the paper, and especially the abstract, gives the impression that the dataset was created specifically for deep learning. I suggest to make it more general for computer-based diagnostics, as the data itself has very little to do with deep learning, and in fact any method can be tested using these data. Such methods can include also automatic model-driven methods that mimic the work of the pathologist, rather than the data-driven deep learning and other related approaches. Deep learning might be a "buzzword" in 2018, but five years from now there might be another buzzword, but the data will probably still be useful and relevant (H&E has been used for many years).Similar statements are also made in the Background section: "To train deep learning models, large, well-curated datasets are needed to both train these models and accurately evaluate their performance". The sentence is logically correct, but such data are required for training any machine learning model, not just deep learning.The claim that "deep learning have opened an avenue…" is an overstatement, as algorithms that are not based on deep learning demonstrated good recognition accuracy in pathology, in fact as early as the 1990's, without using deep learning. That whole sentence gives the impression that automatic classification of H&E slides for pathology is a new field, while it clearly isn't. I therefore recommend to weaken the statement or make it more general to machine learning. It seems to me that the term "deep learning" is confused with the term "machine learning".Page 3: The image file format is discussed (TIFF), but without important details. What is the resolution of the images? What is the dynamic range? Bits per pixel? Channels per pixel? Data type (integer, floating point)? etc. The data annotation process is not entirely clear. Pathology can be subjective and different pathologists might reach different conclusions regarding the same slide. The important information about the data annotation is a little vague. For instance, what was the disagreement rate between the pathologists in the different stages? In how many of the cases the inspection by the breast cancer pathologist (PB or PvD) led to a change in the label? I have some painful experience with benchmark dataset that did not really reflect just the real-world problem they were collected for. http://onlinelibrary.wiley.com/doi/10.1111/j.1365-2818.2011.03502.x/abstracthttps://link.springer.com/article/10.1007%2Fs11263-008-0143-7http://ieeexplore.ieee.org/document/7299607/?reload=true&arnumber=7299607These can be most risky when using deep learning, where the features are not intuitive and the only practical way to validate the reliability of the results is careful design of the dataset and sound controls. Apparently, such algorithms can identify the imaging device, and in some cases even the technician acquiring the images, sometimes leading to good prediction accuracy achieved without solving the original problem (as shown in the links above). Therefore, it is not uncommon that models show good accuracy when using the same dataset separated to training and test data, but much lower accuracy when tested with data from a different set. That can even happen with images collected from the internet:http://ieeexplore.ieee.org/document/5995347/The dataset described in this paper combines data from multiple medical centers and using different imaging devices,

which is good. However, the dataset is still based on a fixed number of sources, and therefore algorithms showing good performance might still be limited to the specific data used in the dataset, and there is no guarantee that the same algorithm performs well also on data from sources it had not "seen" and trained with. As I proposed in the past, one way of solving a problem of this kind is to use data acquired from one center for training, and data from a different center for testing. Good results achieved using this experimental design indicate that the algorithm is not limited to a certain dataset. From the paper it seems that data from all centers were used for both training and testing, and therefore the current design does not test whether a model trained with the dataset can also annotate data coming from other centers that are not included in the dataset. I understand that after the grand challenge has already started and teams have already submitted their results it will be difficult to make a change in the design. However, a clear discussion about that limitation should be added. My understanding is that even with the current data, if researchers are aware of the issue they can separate the data into different centers and perform such experiment, testing how their algorithm performs on data from a center not used for training data.The dataset is organized in the form of a grand challenge (like Kaggle, for instance), in which the authors do not release the annotation of the test data, but serve as the judges for teams that submit their results. The evaluation is done on the backend, and without the participation of the teams. The scientific motivation behind that practice should be discussed and explained. Kaggle is a very good service, and the practice of a competition is common in pattern recognition (e.g., ImageNet), but in the context of cancer diagnostics the impact and optimization of scientific return through the form of a grand challenge should be explained. The fact that it is a grand challenge should also be mentioned in the abstract.In the context of that grand challenge, I was looking to find some description of how the results are evaluated, but did not find any information. There is indeed some information in the web site, but the information should also be given in the paper.Page 4, line 52. The paragraph is a repetition of the previous section.Page 6: "The dataset has also been used by companies experienced in machine learning application to be a ¬first foray into digital pathology, for example Google [22]." How is reference 22 related to Google?

Level of Interest

Please indicate how interesting you found the manuscript: An article of importance in its field

Quality of Written English

Please indicate the quality of language in the manuscript: Acceptable

Declaration of Competing Interests

Please complete a declaration of competing interests, considering the following questions:

- Have you in the past five years received reimbursements, fees, funding, or salary from an organisation that may in any way gain or lose financially from the publication of this manuscript, either now or in the future?
- Do you hold any stocks or shares in an organisation that may in any way gain or lose financially from the publication of this manuscript, either now or in the future?
- Do you hold or are you currently applying for any patents relating to the content of the manuscript?
- Have you received reimbursements, fees, funding, or salary from an organization that holds or has applied for patents relating to the content of the manuscript?
- Do you have any other financial competing interests?

- Do you have any non-financial competing interests in relation to this paper?

If you can answer no to all of the above, write 'I declare that I have no competing interests' below. If your reply is yes to any, please give details below.

I declare that I have no competing interests

I agree to the open peer review policy of the journal. I understand that my name will be included on my report to the authors and, if the manuscript is accepted for publication, my named report including any attachments I upload will be posted on the website along with the authors' responses. I agree for my report to be made available under an Open Access Creative Commons CC-BY license (http://creativecommons.org/licenses/by/4.0/). I understand that any comments which I do not wish to be included in my named report can be included as confidential comments to the editors, which will not be published.

I agree to the open peer review policy of the journal

To further support our reviewers, we have joined with Publons, where you can gain additional credit to further highlight your hard work (see: https://publons.com/journal/530/gigascience). On publication of this paper, your review will be automatically added to Publons, you can then choose whether or not to claim your Publons credit. I understand this statement.

Yes
