# Supplementary material for: 1399 H&E-stained sentinel lymph node sections of breast cancer patients: the CAMELYON dataset
Source: Gigascience. 2018 May 31;7(6):giy065. doi: 10.1093/gigascience/giy065 (PMC6007545; doi:10.1093/gigascience/giy065)
Supplement: Reviewer_2_Report_(Original_Submission) -- Chris Armit — 1/4/2018 Reviewed [file giy065_reviewer_2_report_(original_submission).pdf]

Reviewer Report

Title: 1399 H&E-stained sentinel lymph node sections of breast cancer patients: the CAMELYON dataset

Version: Original Submission **Date:** 1/4/2018

Reviewer name: Chris Armit

Reviewer Comments to Author:

In this Data Note, the authors describe a large morphological study of digitised lymph node sections that could be used for exploring the ability of machine-learning algorithms to identify metastases on tissue sections. The lymph node specimens were collected from 5 different medical centres and the histopathological status was scored using TNM staging criteria. In the first study (CAMELYON16), a lab technician and a PhD student performed staging and expert pathologists confirmed the annotations. In a second study (CAMELYON17), a general pathologist staged the lymph node specimens, and detailed annotations were validated by one of two pathology residents. In addition, the authors describe the publicly available Automated Slide Analysis Platform (ASAP) software package that can be used to view whole-slide images, annotations and algorithmic results. The manuscript is well-written and I consider the CAMELYON dataset of great interest to the machine-learning community.Major issue 1The CAMELYON dataset is available under Creative Commons License CC-BY-NC-ND. This implies that the data is free to share for non-commercial use. However, with this current license agreement the CAMELYON dataset may not be used for commercial purposes. Furthermore, the CC-BY-NC-ND license agreement implies that derivatives from these material, which could include segmentations of the original image data, may not be distributed commercially or non-commercially. This severely impinges on the utility of this dataset for machine-learning. The authors should consider changing the Creative Commons License agreement for the CAMELYON dataset so that re-use is encouraged.Minor issue 1I would like more detail on how the polygon tool was used to manually delineate metastases. In particular, could the authors provide details of whether the immunohistochemically-labelled slides stained with anti-cytokeratin were used as a guide for annotating the adjacent H&E sections? Alternatively, were the H&E sections labelled directly without first inspecting the cytokeratin-labelled sections? Minor issue 2In addition, it would be good to know whether a consensus was reached between multiple pathologists in validating the hand-drawn annotations as this may impact on the ability of machine-learning algorithms to computationally identify metastases. Was there a consensus between multiple pathologists for all 399 hand-drawn contours produced from the CAMELYON16 dataset? Similarly, was there a consensus between multiple pathologists for all 50 hand-drawn contours that were produced from the CAMELYON17 dataset?Minor issue 3Details of the primary and secondary antibodies used to stain for pan-cytokeratin have not been provided. If the various different medical centres used different antibodies, then this should be clearly stated in the manuscript as it may impact on the ability of machine-learning algorithms to process the immunohistochemically-labelled image data.Minor issue 4Figure 4 shows the tissue mask overlay at low-resolution and it is very difficult to see how accurate the mask overlays the lymph node tissue. The authors should consider revising this figure to include higher-resolution images so that the mask overlay is clearly seen.

Level of Interest

Please indicate how interesting you found the manuscript: An article of importance in its field

Quality of Written English

Please indicate the quality of language in the manuscript: Acceptable

Declaration of Competing Interests

Please complete a declaration of competing interests, considering the following questions:

- Have you in the past five years received reimbursements, fees, funding, or salary from an organisation that may in any way gain or lose financially from the publication of this manuscript, either now or in the future?
- Do you hold any stocks or shares in an organisation that may in any way gain or lose financially from the publication of this manuscript, either now or in the future?
- Do you hold or are you currently applying for any patents relating to the content of the manuscript?
- Have you received reimbursements, fees, funding, or salary from an organization that holds or has applied for patents relating to the content of the manuscript?
- Do you have any other financial competing interests?
- Do you have any non-financial competing interests in relation to this paper?

If you can answer no to all of the above, write 'I declare that I have no competing interests' below. If your reply is yes to any, please give details below.

I declare that I have no competing interests

I agree to the open peer review policy of the journal. I understand that my name will be included on my report to the authors and, if the manuscript is accepted for publication, my named report including any attachments I upload will be posted on the website along with the authors' responses. I agree for my report to be made available under an Open Access Creative Commons CC-BY license (http://creativecommons.org/licenses/by/4.0/). I understand that any comments which I do not wish to be included in my named report can be included as confidential comments to the editors, which will not be published.

I agree to the open peer review policy of the journal

To further support our reviewers, we have joined with Publons, where you can gain additional credit to further highlight your hard work (see: https://publons.com/journal/530/gigascience). On publication of this paper, your review will be automatically added to Publons, you can then choose whether or not to claim your Publons credit. I understand this statement.

Yes
