# Supplementary material for: 1399 H&E-stained sentinel lymph node sections of breast cancer patients: the CAMELYON dataset
Source: Gigascience. 2018 May 31;7(6):giy065. doi: 10.1093/gigascience/giy065 (PMC6007545; doi:10.1093/gigascience/giy065)
Supplement: Reviewer_2_Report_(Revision_1) -- Chris Armit — 02-04-2018 Reviewed [file giy065_reviewer_2_report_(revision_1).pdf]

Reviewer Report

Title: **1399 H&E-stained sentinel lymph node sections of breast cancer patients: the CAMELYON dataset**

Version: **Revision 1** **Date:** 4/2/2018

Reviewer name: **Chris Armit**

Reviewer Comments to Author:

I am grateful to the authors for addressing my questions. In particular, the authors are to be commended for changing the Creative Commons License agreement for the CAMELYON dataset to CC0.In addition, I am grateful to the authors for explaining that annotation was applied to the histopathology H&E sections, and not the adjacent immunohistochemically-labelled sections. This enables a more clear understanding of the annotation procedure that was used in the CAMELYON studies. As the immunohistochemically-labelled sections were used as a guide to identify corresponding areas, I do see great value in the author's provision of the requested antibody details. I suggest that these should be included in the GigaDB page that accompanies the GigaScience manuscript.Furthermore, I am additionally grateful to the authors for explaining why they did not obtain consensus annotations from multiple pathologists. The authors report that a consensus approach would have been prohibitively costly for a study of this magnitude. In this respect, the machine-learning algorithm-based method outlined here represents a powerful method by which the biomedical community may receive a cost-effective alternative to consensus from a panel of pathologists. The image analysis of CAMELYON16 and CAMELYON17 datasets are an outstanding example of how machine-learning algorithms can be applied to lymph node section data. I anticipate that the alternative pathology workflow outlined in this study will prove invaluable for future prospective histopathology studies.Finally, I thank the authors for providing a higher resolution image for Figure 4. The new image highlights a zoomed in view of the overlay that it is much easier to interpret.

Level of Interest

Please indicate how interesting you found the manuscript: An article of importance in its field

Quality of Written English

Please indicate the quality of language in the manuscript: Acceptable

Declaration of Competing Interests

Please complete a declaration of competing interests, considering the following questions:

- Have you in the past five years received reimbursements, fees, funding, or salary from an organisation that may in any way gain or lose financially from the publication of this manuscript, either now or in the future?
- Do you hold any stocks or shares in an organisation that may in any way gain or lose financially from the publication of this manuscript, either now or in the future?
- Do you hold or are you currently applying for any patents relating to the content of the manuscript?

- Have you received reimbursements, fees, funding, or salary from an organization that holds or has applied for patents relating to the content of the manuscript?
- Do you have any other financial competing interests?
- Do you have any non-financial competing interests in relation to this paper?

If you can answer no to all of the above, write 'I declare that I have no competing interests' below. If your reply is yes to any, please give details below.

I declare that I have no competing interests.

I agree to the open peer review policy of the journal. I understand that my name will be included on my report to the authors and, if the manuscript is accepted for publication, my named report including any attachments I upload will be posted on the website along with the authors' responses. I agree for my report to be made available under an Open Access Creative Commons CC-BY license (http://creativecommons.org/licenses/by/4.0/). I understand that any comments which I do not wish to be included in my named report can be included as confidential comments to the editors, which will not be published.

I agree to the open peer review policy of the journal

To further support our reviewers, we have joined with Publons, where you can gain additional credit to further highlight your hard work (see: https://publons.com/journal/530/gigascience). On publication of this paper, your review will be automatically added to Publons, you can then choose whether or not to claim your Publons credit. I understand this statement.

Yes
